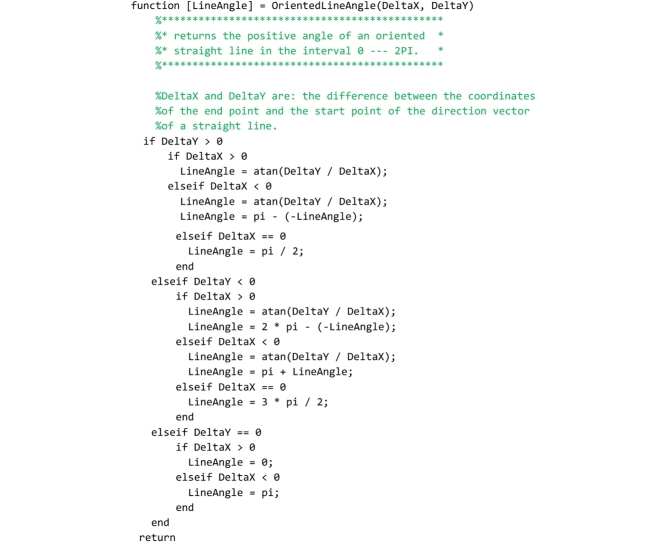# The Matlab code of the method based on the Full Range Factor for assessing the safety of masonry arches

**DOI:** 10.1016/j.mex.2019.05.033

**Published:** 2019-06-04

**Authors:** Stefano Galassi, Giacomo Tempesta

**Affiliations:** Department of Architecture, University of Florence, Piazza Brunelleschi 6, 50121 Florence, Italy

**Keywords:** Full Range Factor of Safety Method (FRS Method), Discrete arches, Line of thrust, Finite differences, Safety assessment, Heyman, Geometrical factor, Performance factor, Matlab code

## Abstract

In most masonry arches stresses are very low and, therefore, collapse does not occur because of material failure. As a consequence, the safety of arches should not be assessed by means of a safety factor based on material strength as for conventional structures. In 1969 Heyman was the first to state that the safety of masonry arches relies on their geometry and proposed a method for computing the so-called “geometrical factor of safety” based on the comparison between the shape of the thrust line and the profile of the arch. In this context, we have recently developed a method capable of computing the line of thrust closest to the geometrical axis and defining a safety factor based on the comparison between such a line of thrust and the profile of the arch, which we have denoted as “performance factor”. In this paper, that supplements the author ref. (Tempesta and Galassi, 2019 [41]), the Matlab code of our method is provided for unlimited and unrestricted use by researchers as well as academics for educational purposes.

• The method (denoted as FRS Method) is inspired by the method proposed by Heyman in 1969
• Unlike the original iterative method, the FRS Method computes the line of thrust using a one-step procedure, which is less time consuming and provides the exact solution
• The original geometrical factor of safety is replaced by a performance factor, that characterizes the range of the equilibrium thrust lines within the profile of the arch effectively and the safety factor in a targeted way

## Graphical abstract

**Figure:**
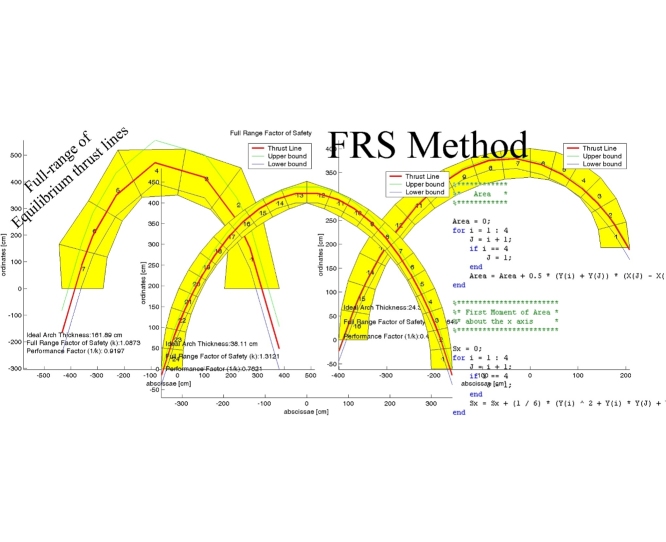

**Specifications Table**

**Table:** 

Subject Area:	*Engineering*
More specific subject area:	*Safety assessment of masonry arches based upon their geometry*
Method name:	*Full Range factor of Safety Method (FRS Method)*
Name and reference of original method	*The original method by Heyman* [1] *computes the line of thrust closest to the geometrical axis of an arch using an iterative method that proceeds by trial and error. The safety of the structure is successively assessed by defining the thickness of an ideal arch (i.e. the arch of minimal thickness) within the profile of the real arch (the geometrical factor of safety).* [1] *J. Heyman, The safety of masonry arches, Int. J. Mech. Sci. (1969) 11(4): 363-85.*
Resource availability	[41] *G. Tempesta, S. Galassi, Safety evaluation of masonry arches. A numerical procedure based on the thrust line closest to the geometrical axis, Int. J. Mech. Sci. (2019) 155: 206–21.*

## Method details

### Background

Collapses of masonry arches which occurred in past times clearly demonstrated that their geometrical profile is the main feature responsible for safety. Based on this, Heyman [1] proposed an iterative method for computing the line of thrust closest to the geometrical axis and pointed a factor for assessing the safety, which relied exclusively on geometrical considerations. Such a factor, denoted as “geometrical factor of safety”, was defined as the ratio between the actual thickness of the arch and the thickness of the minimal arch within the profile of the real one, obtained by scaling its thickness to enclose the thrust line. Thenceforth, the scientific community has shared the Heymanian school of thinking and his theory has been used to assess the safety of masonry arches and vaults in the context of limit or incremental analysis [[2], [3], [4], [5], [6], [7], [8], [9], [10], [11], [12], [13], [14], [15], [16], [17], [18], [19], [20], [21], [22], [23], [24], [25], [26], [27], [28]].

Limit analysis is used to assess the load-carrying capacity and the safety level, avoiding the mechanical properties of materials to be estimated [29,30] as is required by non-linear elastic incremental analyses [[31], [32], [33], [34], [35], [36], [37]], through which the “exact” solution and the “true” line of thrust can be achieved, but long iterative analyses are needed. In the literature, methodologies and computer programs based on the kinematic theorem [5,14,17], denoted as “mechanism methods”, or on the static theorem [3,4,6,15], denoted as “thrust line methods”, have been proposed and have largely replaced the earlier hand based techniques, such as the famous Mery’s method [38].

Generally, mechanism methods assume that a masonry arch becomes a mechanism when at least four hinges occur. However, hinge position is unknown and procedures based on these methods must assume trial positions and perform several computations, using the equilibrium equations at the hinges [5] or the equations of virtual work [14]. Since the theorems of limit analysis do not provide unique solutions for the collapse load factor if a non-associative friction rule is assumed [39], in order to also take into account sliding mechanisms due to finite friction, robust numerical procedures have been formulated [6,18,19,25], but they usually use a static equilibrium approach.

Thrust line methods, instead, assess the safety level using procedures that compute the line of thrust by solving the equilibrium equations or a linear programming problem and identify the zone where the inner forces (i.e. the thrust line) can stand. This zone is a domain that has been defined in the literature according to the middle third rule, which is derived from the elastic theory, or the middle half rule, which is a less conservative approach. Lastly, Heyman [1] proposed a revisited version of the aforementioned rules considering that the thrust line can lie within the whole thickness of the arch and, therefore, removed the boundaries of the middle third rule or the half middle rule of the thickness. This criterion is the less conservative one and, indeed, it corresponds to the limit equilibrium condition of an arch and has also been extended to the analysis of vaults [[26], [27], [28]]. All these variants of the thrust line method can be summarized [40] by the Heymanian concept of geometric factor of safety mentioned above, hereafter referred to as the “GFS Method”. In order to overcome limitations of the Heymanian geometric factor of safety for practical use in case of irregular profiles, in [20] the domain of safety has been redesigned as the locus of admissible positions of poles in the force diagrams leading to thrust lines that lie entirely within the masonry envelope and a safety indicator has been pointed out based on the value of the maximum and minimum admissible thrusts.

The authors of this paper, instead, have developed a new method (denoted as “Full Range factor of Safety-based Method”, hereafter referred to as the “FRS Method”) that improves the original method proposed by Heyman in 1969. The FRS Method is described in detail in [41] and this paper, which provides a brief technical description of it in the next two sections and the full Matlab code in the following pages, supplements author’s ref. [41]. Readers can use and modify these Matlab routines without restrictions for research and educational purposes. Conversely, professional use of this code is forbidden and the authors will not be responsible for it.

Unlike the original method proposed by Heyman, that is an iterative method that computes the line of thrust closest to the geometrical axis proceeding by trial and errors, the FRS Method is a one-step procedure. Thus, this new method allows obtaining the “exact” solution of the problem and a reduction in time is assured. Furthermore, a new domain of safety has been conceived and obtained by shifting the thrust line vertically until it touches the extrados and intrados profiles of the arch. This domain of safety, that has been denoted as “full-range of equilibrium thrust lines”, provides a safety indicator (the performance factor) computed as the ratio between the vertical thickness of the domain and the minimum vertical thickness of the arch.

This procedure applies to any profile of the arch, subject only to vertical forces. In [41] the inverted catenary-shaped arch, the circular arch, the segmental arch, the pointed arch, the rampant arch and also an arch of generic shape have been analyzed. In the current formulation, the procedure cannot analyze multi-ring nor multi-span arches. At present, we are developing a new release of the method in order to also account for the horizontal actions provoked by an earthquake and the extension of the method will be capable of assessing the safety of 3D-structures such as barrel and cross vaults.

### Brief description of the method

**Fig. 1:**
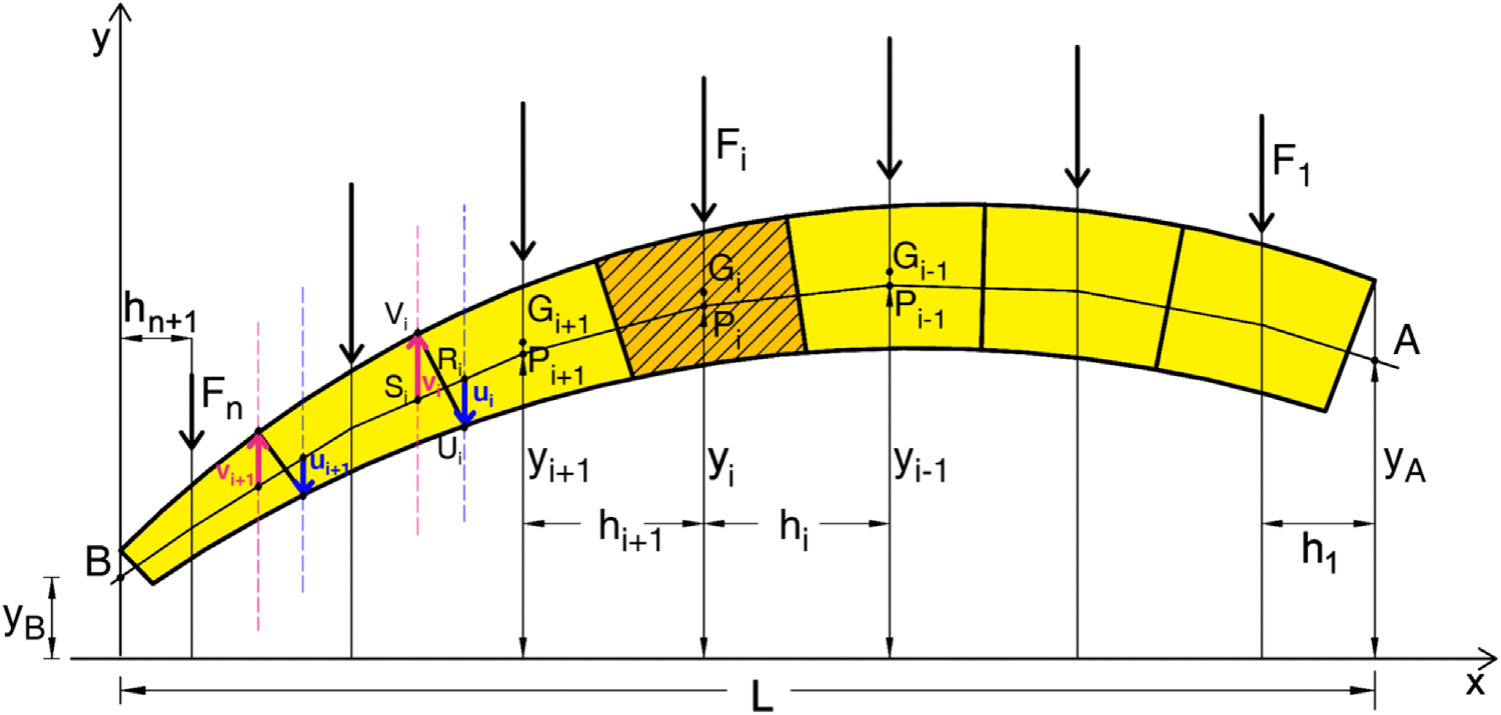
The arch discrete model (adapted from [41]).

The arch is regarded as a continuous structure but, in order to allow the method to be generalized to all geometrical profiles and load conditions, it is conveniently divided into a finite number *n* of discrete elements (Fig. 1). In so doing, the analysis of a rigid block arch is also allowed, matching the blocks with the elements and the joints between blocks with the lines of separation between them.

Hereafter, the main steps of the method are listed, although for a more in-depth understanding of the numerical procedure we suggest that the interested reader examines the main paper [41]:

1 For each discrete element *i* of the arch the position of the centroid G_i_ and the vertical load F_i_ acting on it are computed;
2 In order to identify a specific line of thrust (that closest to the geometrical axis, as conceived in [42]) among the ∞^3^ likely lines of thrust in equilibrium with the loads, three parameters or conditions have been imposed: the ordinate Y_0_ of point A and the ordinate Y_n+1_ of point B, for which the first and the last segments of the thrust line must pass, and the horizontal component of the thrust H;
3 To ensure that the line of thrust identified at the previous step is exactly the one closest to the geometrical axis, the distances d_i_ = Y_i_ − Y_Gi_ among the vertices P_i_ of the line of thrust and the centroids G_i_ that define the geometrical axis are minimized.

The minimization procedure is written in algebraic form and makes use of the finite difference method. Therefore, the system of the equilibrium equations of the internal and external forces acting on each vertex of the line of thrust is written (Eq. (1)):

(1) -1h1·Y0+h1+h2h1∙h2·Y1+-1h2·Y2=F1H-1h2·Y1+h2+h3h2∙h3·Y2+-1h3·Y3=F2H………………………………………………..-1hi·Yi-1+hi+hi+1hi∙hi+1·Yi+-1hi+1·Yi+1=FiH………………………………………………..-1hn·Yn-1+hn+hn+1hn∙hn+1·Yn+-1hn+1·Yn+1=FnH

The unknowns of the system in Eq. (1) are the *n+2* ordinates Y_i_ of vertices of the line of thrust and the thrust H. Therefore, it is indeterminate to three degrees and to solve it the three conditions mentioned at step 2 are imposed. For this purpose, putting K = H^−1^, the system is rearranged isolating the three redundant unknowns (Eq. (2)):

(2) $$\left[\begin{array}{cccc} \left(\frac{{\text{h}}_{1}+{\text{h}}_{2}}{{\text{h}}_{1}∙{\text{h}}_{2}}\right) & \left(\frac{-1}{{\text{h}}_{2}}\right) & 0 & 0\\ \left(\frac{-1}{{\text{h}}_{2}}\right) & \left(\frac{{\text{h}}_{2}+{\text{h}}_{3}}{{\text{h}}_{2}∙{\text{h}}_{3}}\right) & \left(\frac{-1}{{\text{h}}_{3}}\right) & 0\\ \ldots & \ldots & \ldots & \ldots \\ 0 & \left(\frac{-1}{{\text{h}}_{\text{i}}}\right) & \left(\frac{{\text{h}}_{\text{i}}+{\text{h}}_{\text{i}+1}}{{\text{h}}_{\text{i}}∙{\text{h}}_{\text{i}+1}}\right) & \left(\frac{-1}{{\text{h}}_{\text{i}+1}}\right)\\ \ldots & \ldots & \ldots & \ldots \\ 0 & 0 & \left(\frac{-1}{{\text{h}}_{\text{n}}}\right) & \left(\frac{{\text{h}}_{\text{n}}+{\text{h}}_{\text{n}+1}}{{\text{h}}_{\text{n}}∙{\text{h}}_{\text{n}+1}}\right) \end{array}\right]\cdot \left\{\begin{array}{c} {\text{Y}}_{1}\\ {\text{Y}}_{2}\\ \ldots \\ {\text{Y}}_{\text{i}}\\ \ldots \\ {\text{Y}}_{\text{n}} \end{array}\right\}=\left\{\begin{array}{c} {\text{F}}_{1}\\ {\text{F}}_{2}\\ \ldots \\ {\text{F}}_{\text{i}}\\ \ldots \\ {\text{F}}_{\text{n}} \end{array}\right\}\cdot \text{K}+\left\{\begin{array}{c} 1/{\text{h}}_{1}\\ 0\\ \ldots \\ 0\\ \ldots \\ 0 \end{array}\right\}\cdot {\text{Y}}_{0}+\left\{\begin{array}{c} 0\\ 0\\ \ldots \\ 0\\ \ldots \\ 1/{\text{h}}_{\text{n}+1} \end{array}\right\}\cdot {\text{Y}}_{\text{n}+1}$$

and written, more compactly, in matrix form (Eq. (3)):

(3) $$\left[\text{D}\right]\left\{\text{Y}\right\}=\left\{{\text{T}}_{1}\right\}\cdot \text{K}+\left\{{\text{T}}_{2}\right\}\cdot {\text{Y}}_{0}+\left\{{\text{T}}_{3}\right\}\cdot {\text{Y}}_{\text{n}+1}$$

The solution of Eq. (3), provided by Eq. (4), is not computable yet because K, Y_0_, Y_n+1_ are unknown parameters:

(4) $$\left\{\text{Y}\right\}=\left\{{\text{R}}_{1}\right\}\cdot \text{K}+\left\{{\text{R}}_{2}\right\}\cdot {\text{Y}}_{0}+\left\{{\text{R}}_{3}\right\}\cdot {\text{Y}}_{\text{n}+1}$$

In Eq. (4), for clarity of reading, is put: $\left\{{\text{R}}_{1}\right\}={\left[\text{D}\right]}^{-1}\left\{{\text{T}}_{1}\right\}$, $\left\{{\text{R}}_{2}\right\}={\left[\text{D}\right]}^{-1}\left\{{\text{T}}_{2}\right\}$, $\;\left\{{\text{R}}_{3}\right\}={\left[\text{D}\right]}^{-1}\left\{{\text{T}}_{3}\right\}$.

Thus, as the objective of the analysis is the detection of the line of thrust closest to the geometrical axis of the arch, in order to compute the value of the three unknown parameters the function S, which expresses the square of the distances between the vertices of the line of thrust and the centroids of the elements, is minimized (Eq. (5)):

(5) $$\text{S}={\left(\left\{{\text{R}}_{1}\right\}\cdot \text{K}+\left\{{\text{R}}_{2}\right\}\cdot {\text{Y}}_{0}+\left\{{\text{R}}_{3}\right\}\cdot {\text{Y}}_{\text{n}+1}-\left\{{\text{Y}}_{\text{G}}\right\}\right)}^{2}$$

To minimize function S, the conditions that express the zeroing of the three partial derivatives with respect to the three unknown parameters are formulated (Eq. (6)):

(6) $$\begin{array}{l} \frac{\partial \text{S}}{\partial \text{K}}({\text{Y}}_{0},\;{\text{Y}}_{\text{n}+1},\text{K})=0\\ \frac{\partial \text{S}}{\partial {\text{Y}}_{0}}({\text{Y}}_{0},\;{\text{Y}}_{\text{n}+1},\text{K})=0\\ \frac{\partial \text{S}}{\partial {\text{Y}}_{\text{n}+1}}({\text{Y}}_{0},\;{\text{Y}}_{\text{n}+1},\text{K})=0 \end{array}$$

Developing Eq. (6), we obtain the final system of three linear equations (Eq. (7)), whose solution provides the value of the three unknown parameters:

(7) $$\left[\begin{array}{ccc} {\left\{{\text{R}}_{1}\right\}}^{2} & \left\{{\text{R}}_{1}\right\}\left\{{\text{R}}_{2}\right\} & \left\{{\text{R}}_{1}\right\}\left\{{\text{R}}_{3}\right\}\\ \left\{{\text{R}}_{1}\right\}\left\{{\text{R}}_{2}\right\} & {\left\{{\text{R}}_{2}\right\}}^{2} & \left\{{\text{R}}_{2}\right\}\left\{{\text{R}}_{3}\right\}\\ \left\{{\text{R}}_{1}\right\}\left\{{\text{R}}_{3}\right\} & \left\{{\text{R}}_{2}\right\}\left\{{\text{R}}_{3}\right\} & {\left\{{\text{R}}_{3}\right\}}^{2} \end{array}\right]\cdot \left\{\begin{array}{c} \text{K}\\ {\text{Y}}_{0}\\ {\text{Y}}_{\text{n}+1} \end{array}\right\}=\left\{\begin{array}{c} \left\{{\text{R}}_{1}\right\}\left\{{\text{Y}}_{\text{G}}\right\}\\ \left\{{\text{R}}_{2}\right\}\left\{{\text{Y}}_{\text{G}}\right\}\\ \left\{{\text{R}}_{3}\right\}\left\{{\text{Y}}_{\text{G}}\right\} \end{array}\right\}$$

The ordinates of the vertices of the line of thrust are lastly computed substituting backwards such parameters into Eq. (4).

### Full-range factor of safety

Finally, the line of thrust closest to the geometrical axis, obtained using the method above, is used to assess a factor of safety. According to Heyman [1], since arches have low stresses and collapse does not generally occur because of material failure, such a factor can be assessed based on their geometry by simply comparing the shape of the line of thrust to the profile of the arch. The safety factor is identified based on the line of thrust “capacity” of being moved vertically while still remaining contained within the profile of the arch. It is worth noting that, the arch being discretized in a finite number of elements (or blocks), the check of the line of thrust being contained within the profile of the arch must be carried out only in correspondence to the lines of separation among the elements (i.e. the joints of a rigid block), where the points of pressures (points of application of the inner resultant forces) are placed.

The line of thrust is vertically shifted, both upwards and downwards, until it becomes tangent to the extrados and intrados curves of the arch, in such a way to obtain two limit lines of thrust. These lines, denoted as “upper and lower bound thrust lines”, identify a region that describes the domain of the admissible stress states, which is the domain of the admissible lines of thrust parallel to that provided by the procedure (domain of safety).

As reported in [42], to detect the lower (upper) bound of the domain a four step algorithm has been conceived (Fig. 1):

1) The points R_i_ (S_i_) (with i = 1 to n+1) of intersection between the straight lines passing through all the intrados (extrados) point U_i_ (V_i_) of the discrete arch and the thrust line are computed;
2) The vertical vectors **u_i_**  = (U_i_ − R_i_) and **v_i_** = (V_i_ − S_i_) are determined;
3) The vertical distance to which the line of thrust must be shifted to obtain the lower bound of the domain is given by the maximum modulus among all vectors **u_i_** and the vertical distance to which the line of thrust must be shifted to obtain the upper bound of the domain is given by the minimum modulus among all vectors **v_i_**;
4) The vectors ${\left\{{\text{Y}}_{\text{i}\text{n}\text{f}}\right\}=\left\{\text{Y}\right\}+\Delta \text{Y}}_{\text{i}\text{n}\text{f}}$ and ${\left\{{\text{Y}}_{\text{s}\text{u}\text{p}}\right\}=\left\{\text{Y}\right\}+\Delta \text{Y}}_{\text{s}\text{u}\text{p}}$ are lastly computed, whose entries are the ordinates of the points that define the lower and upper lines of thrust respectively, i.e. the lower and upper bound of the domain.

The ratio between the vertical thickness of the arch and the vertical thickness of the domain provides the “full-range factor of safety”, which is a reinterpretation of the “geometrical factor of safety” proposed by Heyman in [1]. Nevertheless, the analyst should assess the safety of an arch referring to the “performance factor”, computed as the reciprocal of the “full-range factor of safety”, because in so doing the range of the factors indicating the degree of safety is comprised between 0 (the lowest safety degree) and 1 (the highest safety degree). Negative factors point out unsafe arches, because they correspond to a negative thickness of the domain of equilibrium thrust lines.

**Table:** 

**Nomenclature of variables**	
Brick	Struct array containing data of the discrete elements of the arch: ‘x(i)’,y’(i)’ are the coordinates of the i-th vertex of the closed polygon that defines the contour of each element (i = 1–4); ‘xG(i)’ and ‘yG(i)’ are the coordinates of the i-th element centroid; ‘Weight’ is the weight of the element and ‘Fy’ is the value of the likely additional vertical force in correspondence to an element.
MaxNumBrick	Is the number of discrete elements.
ResearchLoadFACTOR	If it is set equal to 1 then an additional force Fy in correspondence to an element is assumed to be inputted by the user and the program computes the collapse factor. Otherwise, the value can be set equal to 0 and the analysis is performed for the assigned self-weight loads.
VectorF	Entries of this vector are the horizontal force, vertical force and the moment acting in correspondence to the centroid of each element
OptShape	Struct array containing variables relative to the analysis. If ‘Type_Analysis’ is set by the user equal to 1 then the FRS method is used to compute the safety factor (i.e.: the performance factor); if it is set equal to 2 then the GFS is used (i.e.: the geometrical factor of safety). ‘LoadFactor’ is used by the program and is equal to 1 when only self-weights are present on the arch; increasing values (starting from 1) are used for computing the collapse factor due to an additional increasing load. Entries of vectors 'Vector_Y' and 'Vector_X' are the ordinates and abscissae of the vertices of the line of thrust respectively. Entries of vectors 'Vector_xCPdx', 'Vector_yCPdx', 'Vector_xCPsx' and 'Vector_yCPsx' are the absissae and ordinates of the intersection points between the line of thrust and the right and left joint of each element (i.e. the points of pressure). Entries of variables 'sSUP' and 'sINF' are the superior and inferior distances between the line of thrust and the extrados and intrados curves respectively. ‘sID’ is the overall thickness of the ideal arch (sSUP + sINF), measured along the joint orientation, when the GFS Method is set by the user. Instead, if the FRS Method is set, ‘sID’ measures the vertical thickness of the domain of equilibrium. ‘RealArchMinimumThickness’ is the minimum thickness of all joints. ‘Geom_Safety_Factor’ is the geometrical factor of safety (GFS approach) or the full-range factor of safety (FRS approach). Entries of 'Vector_Ysup' and 'Vector_Yinf' are the ordinates of the upper and lower bounds of the domain of the equilibrium thrust lines if the FRS Method is set by the user. Entry of variable ‘ThrustH’ is the value of the thrust.

### Main routine of the Matlab code

The FRS Method was originally implemented in the software *ArchiVAULT* [43] written both in VisualBASIC 6.0 as for the graphical user interface and in VisualC++ 6.0 as for the numerical computations. *ArchiVAULT* is a complete computer program for performing the analysis of masonry arches developed by the authors. Therefore, with the purpose of providing a stand-alone program specifically dedicated to the assessment of the safety of arches through the FRS Method and freely usable by researchers and academics, routines regarding the FRS Method were translated in Matlab. In this section the Matlab code is provided and discussed. The code is organized in various routines and the routines are saved to separate files. The Matlab language does not distinguish between routines and functions, however, for the sake of clarity, hereafter routines that return a value will be referred to as “functions” and routines that only execute operations will be referred to as “routines”.

The main section of the code that follows must be stored in the “FRS_Method.m” file by the user. In the first lines the set of global variables available for all routines are defined. The nomenclature of the variables is listed above.

**Figure:**
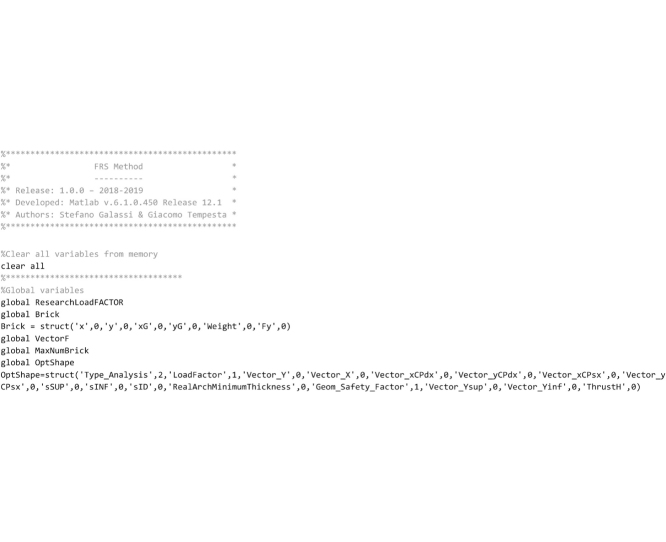

The lines that follow are devoted to the geometrical modeling of the arch, the weight of the elements and the type of analysis to be performed. The user must customize these lines to define a user-defined arch to be analyzed. The parameters set below refers to the Random Arch described in [41].

**Figure:**
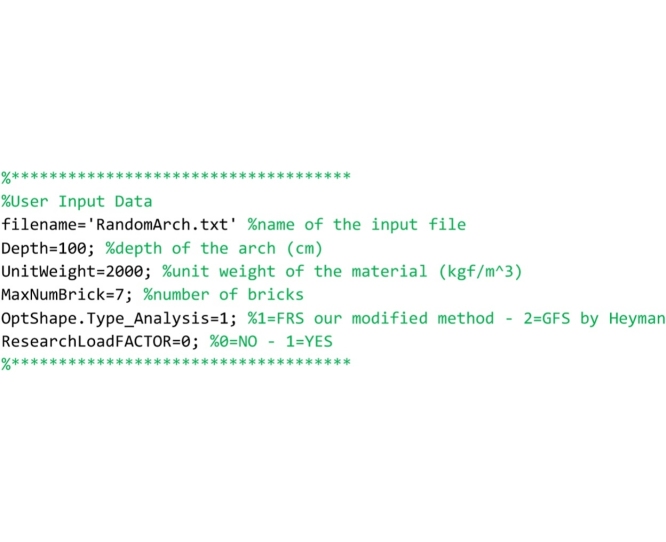

Then the code reads the input TXT file that contains data regarding the shape of the arch and stores the coordinates of the four vertices of each element in the struct vectors Brick(…).x(…) and Brick(…).y(…). The TXT file must be prepared by the user. In the “Input data file” Section the way the user must prepare this file is explained.

**Figure:**
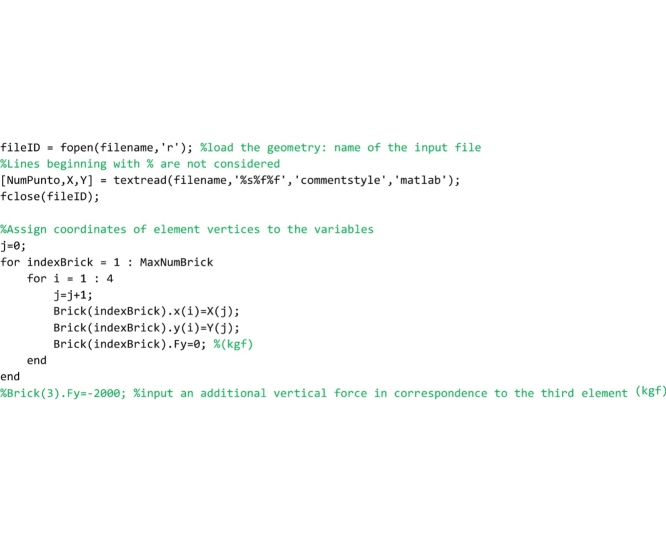

Lines that follow call the ‘ComputeElementCentroid’ routine, that computes the coordinates of the centroid of each element.

**Figure:**
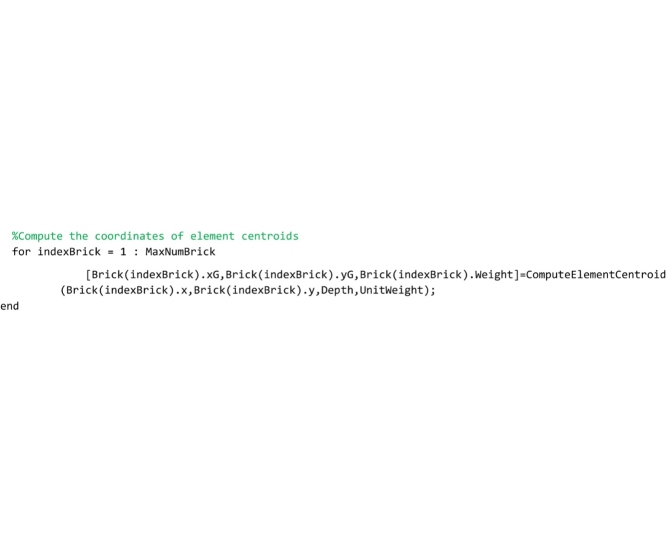

Then the procedure calls the ‘RunAnalysis’ routine, that computes the line of thrust closest to the geometrical axis and, successively, performs the safety assessment through the FRS Method proposed by the authors or through the geometrical factor based method by Heyman (GFS).

**Figure:**
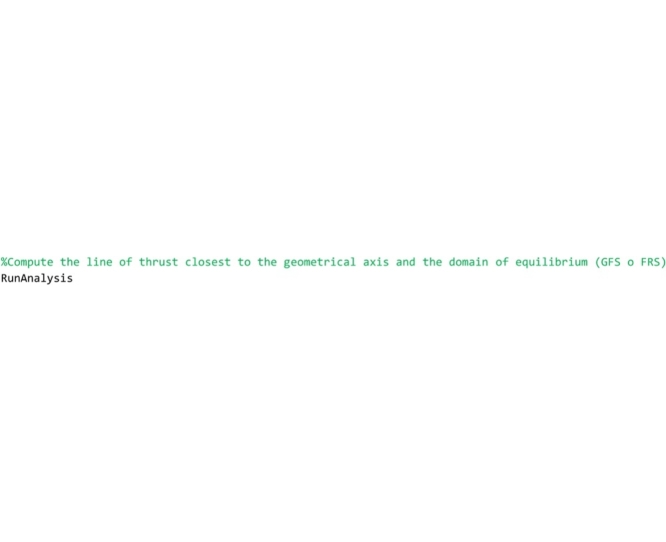

The last part of the code creates a window where the discrete arch is plotted. The line of thrust and the upper and lower bounds of the ideal arch (Heymanian approach) or of the domain of equilibrium thrust lines (our approach) is also plotted on it. The values of the safety factor and the thickness of the ideal arch are also presented in the legend.

**Figure:**
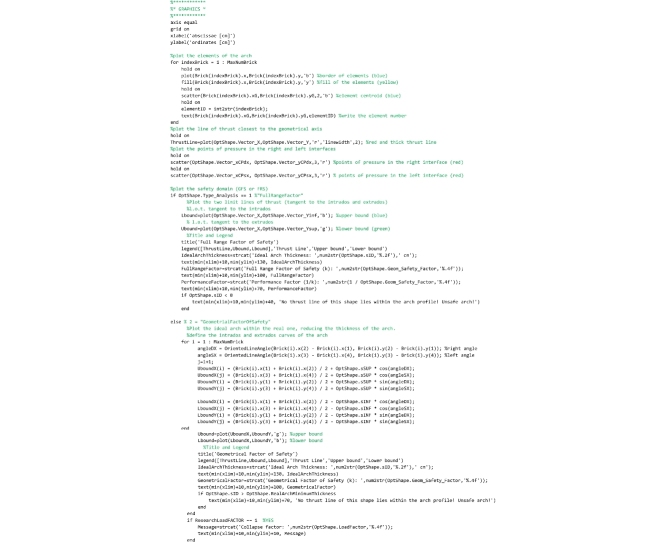

## Input data file

**Fig. 2:**
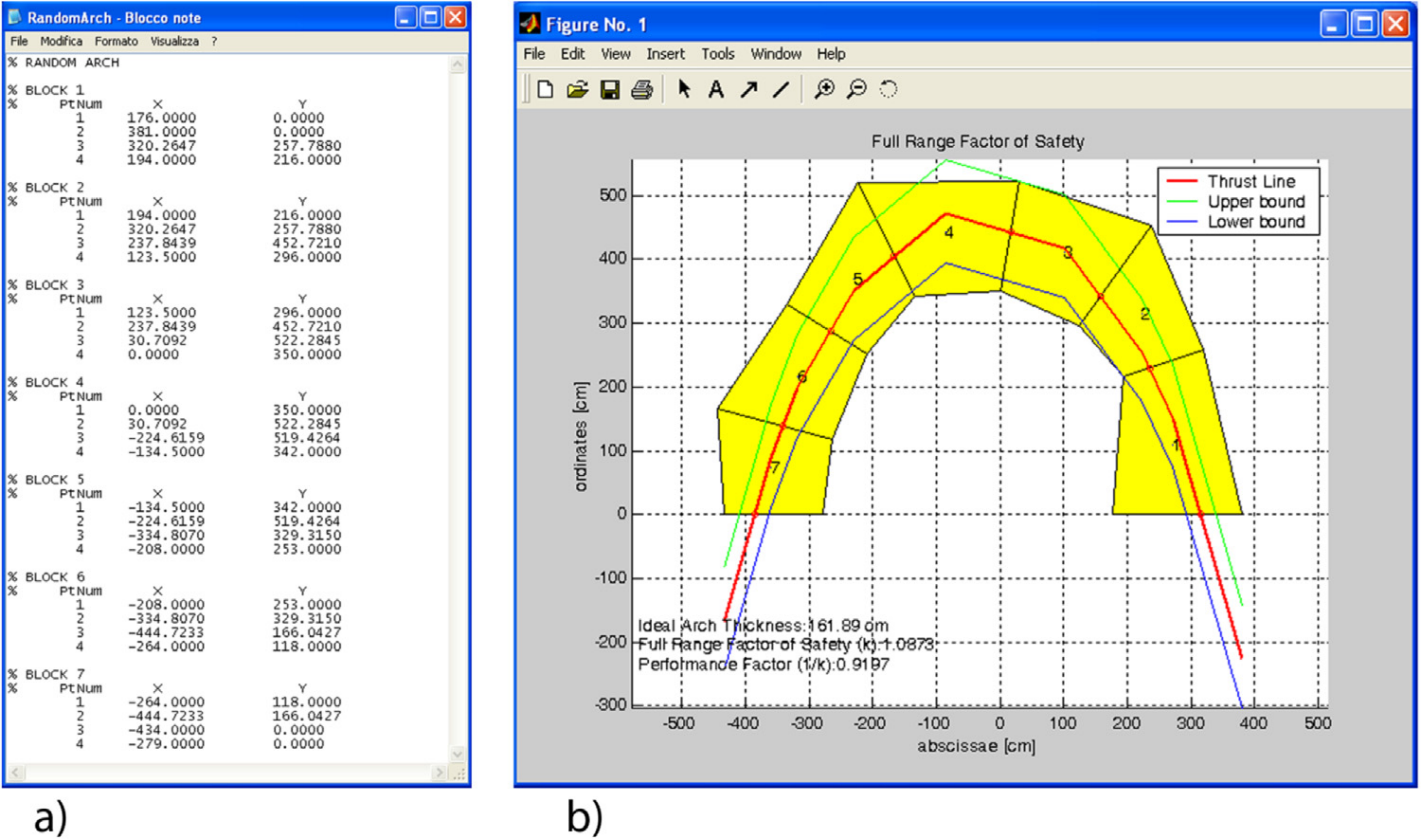
a) Input data file of the Random Arch described in [41]; b) output of results provided by this Matlab code.

By means of a text editor such as NotePad, the user must define a text file (*.txt) for inputting the geometry of the arch in the Matlab program. Fig. 2a shows the text file of the Random Arch, that is a structure subdivided into seven elements. Elements are numbered from right to left and each element is defined as a closed polygon composed of four vertices. Vertices 1 and 2 define the right joint; vertices 3 and 4 define the left joint. Points 1 and 4 are at the intrados of the element and points 2 and 3 are at the extrados. The structure of the text shown if Fig. 2a must be used, in which lines beginning with the ‘%’ character are not read by the Matlab program.

## The “ComputeElementCentroid” function

This function computes the coordinates of element centroids. It must be saved to the ‘ComputeElementCentroid.m’ file by the user. The main routine passes to this function the coordinates of the four vertices of each element, the depth of the arch and the unit weight of the material. Exploiting the Varignon’s theorem, this function returns the element centroid (‘xG’, ‘yG’) and the weight of the element (‘Weight’), by means of a procedure that computes the first moment of area about the x and y axis and the area of the element.

**Figure:**
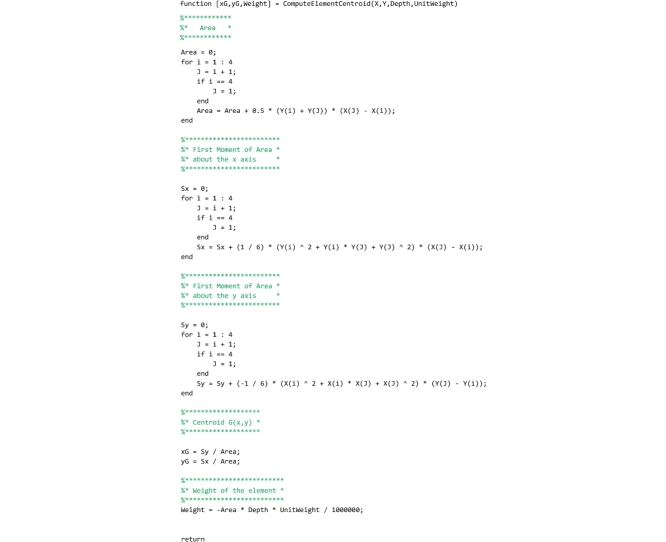

## The “RunAnalysis” routine

This routine, that must be saved to the ‘RunAnalysis.m’ file, runs the analysis and the line of thrust closest to the geometrical axis is computed by calling the BestThrustLine(TempLoadFACTOR) routine. Then, based on the method chosen by the user (FRS or GFS method), the safety verification is performed. If variable OptShape.Type_Analysis is set equal to 1, then the FullRangeFactor routine is called and the domain of equilibrium thrust lines is computed (FRS Method). Instead, if variable OptShape.Type_Analysis is set equal to 2, then the GeomFactor routine is called and the ideal arch is defined (GFS Method). Additionally, this routine also checks if the load factor is required to be computed (this occurs when the TempLoadFACTOR variable has been set equal to 1 by the user). If the load factor is required to be computed, the TempLoadFACTOR variable assumes values greater than 1 and the analysis is rerun iteratively until the limit condition of equilibrium is attained. As an example, Fig. 2b shows the graphical output of the results of the Random Arch, subject to its self-weight, provided by this program.

**Figure:**
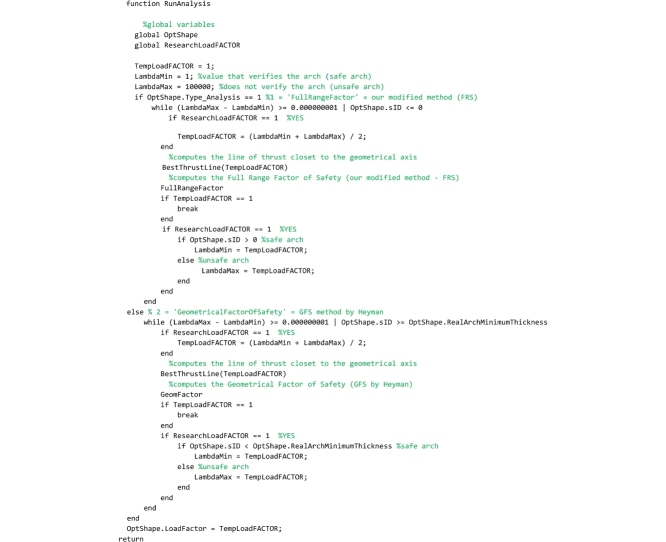

## The “BestThrustLine” routine

This routine computes the line of thrust closest to the geometrical axis and must be saved to the ‘BestThrustLine.m’ file. At the beginning the global variables used herein are declared.

**Figure:**
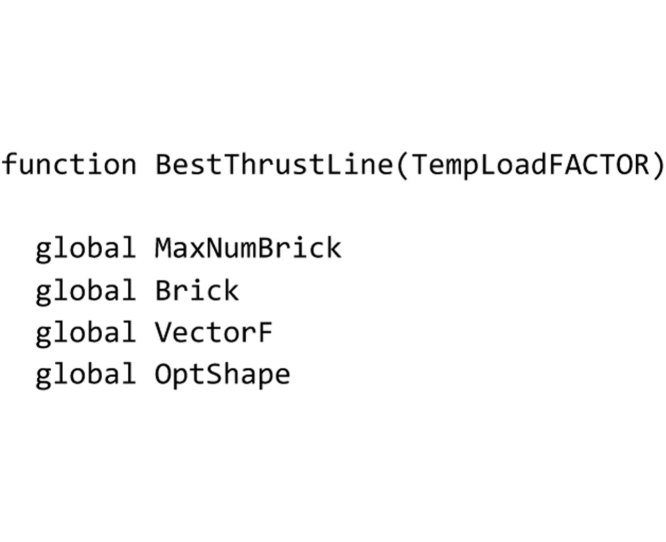

Then, the ComputeLoadVectorF routine, to which the value of the load factor is passed by the TempLoadFACTOR variable, is called.

**Figure:**
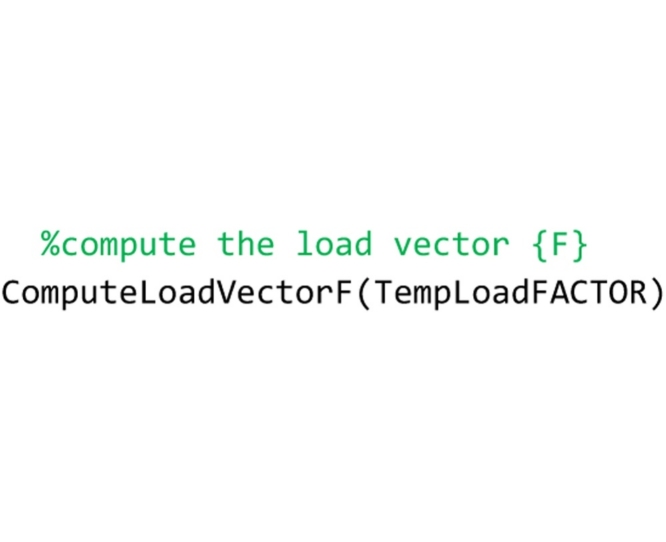

The lines that follow implement the mathematical formulation of the procedure, that builds and solves the system of linear equations that provides the coordinates of the vertices of the line of thrust. The theoretical background of the method is briefly reported in the second section, but a detailed reference of the mathematical procedure is found in [41].

**Figure:**
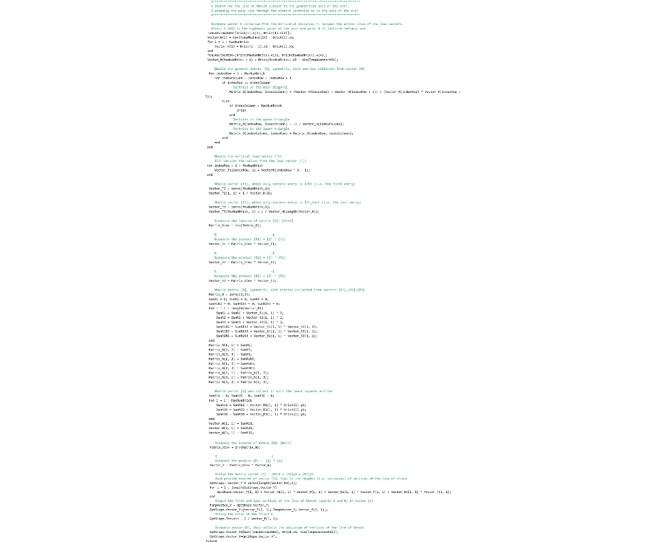

## The “ComputeLoadVectorF” routine

This routine computes the load vector {F} of the structure under analysis. It must be saved to the ‘ComputeLoadVectorF.m’ file by the user. The dimension of the load vector is 3 times the number of elements of the arch, because for each element the horizontal force, the vertical force and the moment as respects to the centroid must be defined. Nevertheless, the current release of the method considers only vertical loads, applied to the element centroids. Therefore, the horizontal force and the moment are zero, while the vertical force is given by the weight of the element plus the value of an additional vertical force, that the user can input in the model when he wants to compute also the load factor.

**Figure:**
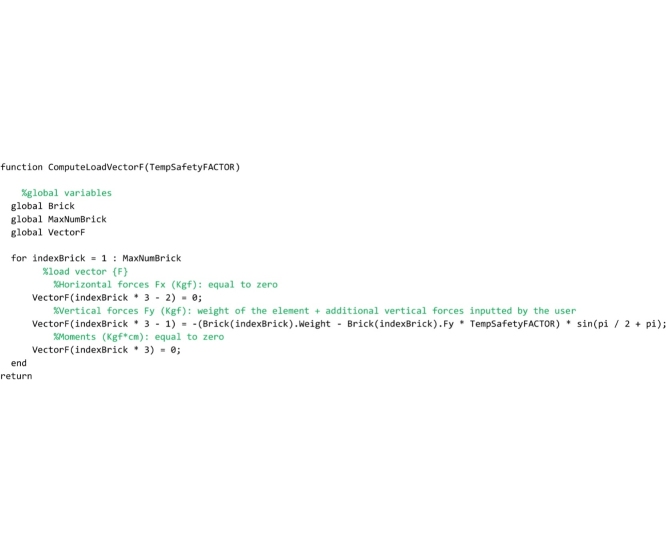

## The “FullRangeFactor” routine

Two routines must be saved to the ‘FullRangeFactor.m’ file. The “FullRangeFactor” routine is the main function, that computes the lower and upper bound of the domain of equilibrium thrust lines and the performance factor. The ‘IntersectionVerticalLine_GenericLine’ function is used to compute the intersection point of two straight lines.

Let us describe the main routine. First the global variables are declared. Then the code calls the ‘IntersectionVerticalLine_GenericLine’ function that computes the intersection points of the left (right) interface and the line of thrust and, as a consequence, returns the points of pressure in the left (right) interface.

**Figure:**
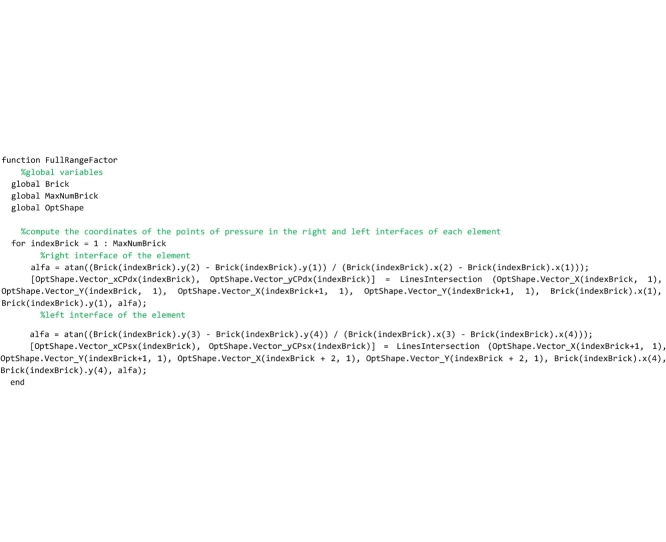

Then, in the following code the lower bound of the domain is computed. The lower bound is defined as that line of thrust obtained by shifting vertically the line of thrust closest to the geometrical axis until it becomes tangent to the intrados curve of the arch. In the following lines of the code, the step by step algorithm for computing the lower bound line of thrust, developed by the authors, is reported and described.

**Figure:**
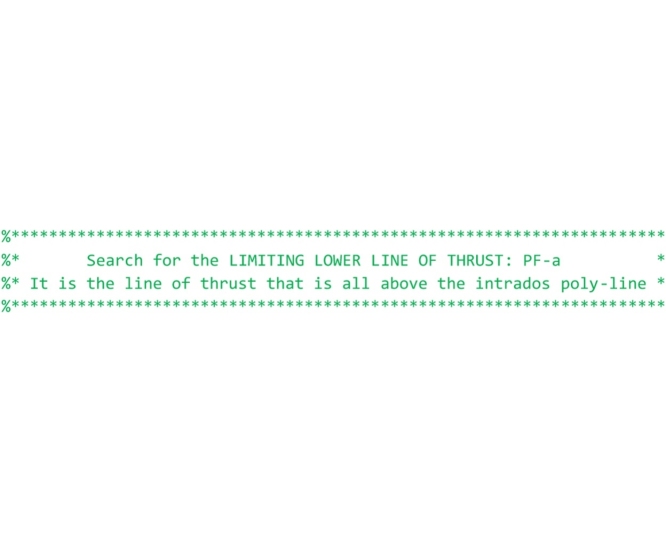

The code that follows searches for the segments of the thrust line that are intercepted by the vertical lines (hereafter referred to as scanning lines) passing through the intrados point of the right interface of each element (point number 1). For the generic scanning line *i*, the segment of the thrust line intercepted by that line is detected by comparing the abscissa of point 1, through which the scanning line passes, and the abscissa of the two end points *i* and *i+1* of each segment of the thrust line. If abscissa of point 1 (referred to as x1 in the code) is comprised between abscissa of point *i+1* and abscissa of point *i*, then the algorithm has found the segment of the thrust line intercepted by the scanning line and the ‘IntersectionVerticalLine_GenericLine’ function is called. This function returns the coordinates of the intersection point of the segment detected and the scanning line, and they are stored in the variables [xK,yK]. Finally, the vertical distance vector between the intersection point K and point 1 are computed and stored in the vector Vector_dINF. This check is repeated testing all segments of the thrust line (for i = 1: length(OptShape.Vector_Y)-1) on the i-th scanning line and the loop is performed for all the scanning lines.

**Figure:**
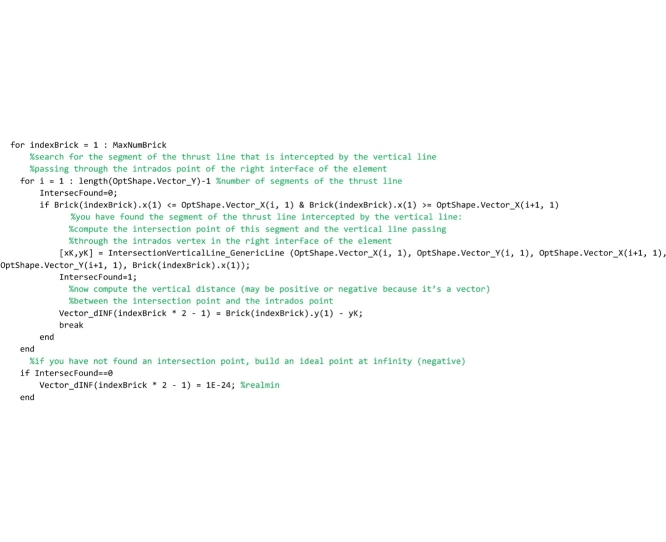

The procedure above is repeated for detecting also the segments of the thrust line that are intercepted by the vertical lines passing through the intrados point of the left interface of each element (point number 4).

**Figure:**
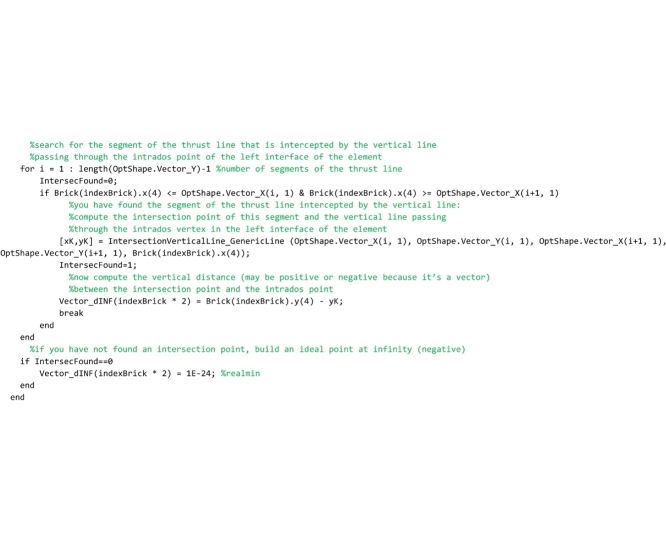

Finally, lines that follow search for the minimum distance vector, among all the distances that have been stored in the vector Vector_dINF.

**Figure:**
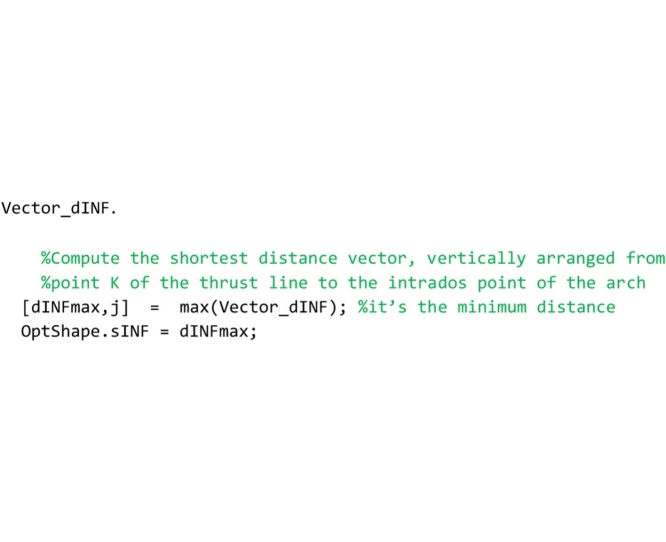

In the following lines, the minimum distance is used to compute the ordinates of the lower bound thrust line that are stored in the global vector OptShape.Vector_Yinf.

**Figure:**
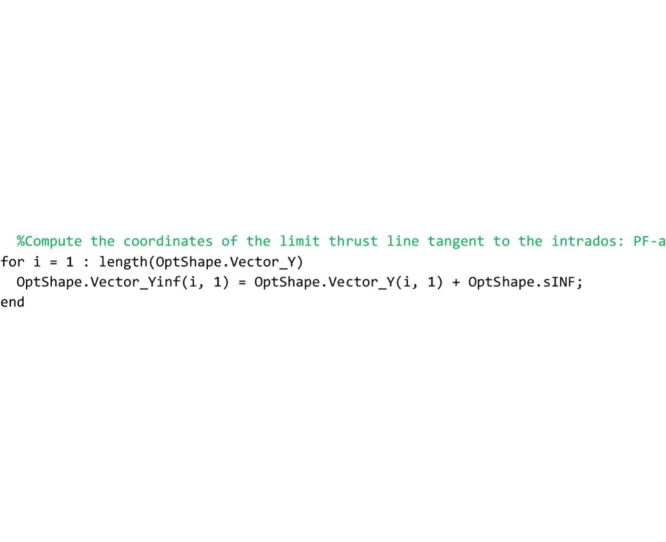

Then, in the following code the upper bound of the domain is computed. Coherently with the lower bound defined above, the upper bound is defined as that line of thrust obtained by shifting vertically the line of thrust closest to the geometrical axis until it becomes tangent to the extrados curve of the arch. Therefore, the scanning lines are defined to pass through the extrados points of the arch (points 2 and 3). The following lines that compute the upper bound line of thrust are identical to the code described above and, therefore, it is not necessary to comment on them.

**Figure:**
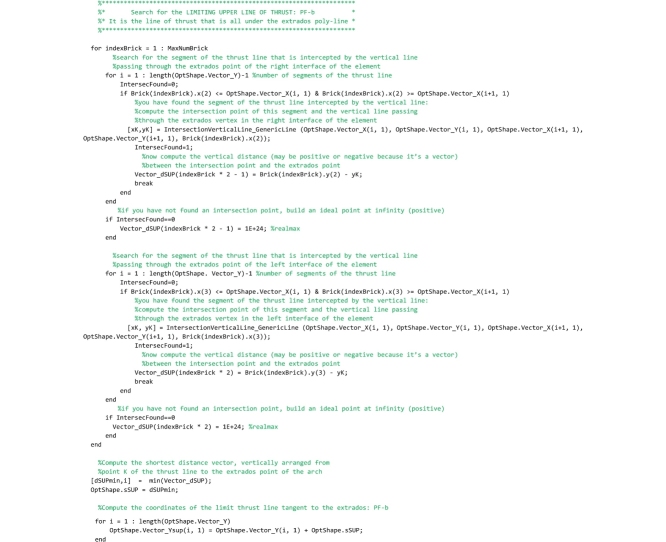

Line that follows computes the minimum thickness of the domain (vertical distance between the lower and upper bound thrust lines) and stores it to the OptShape.sID variable. If the thickness is positive, the domain exists and the arch is safe; if it is negative the domain does not exist and the arch is unsafe.

**Figure:**
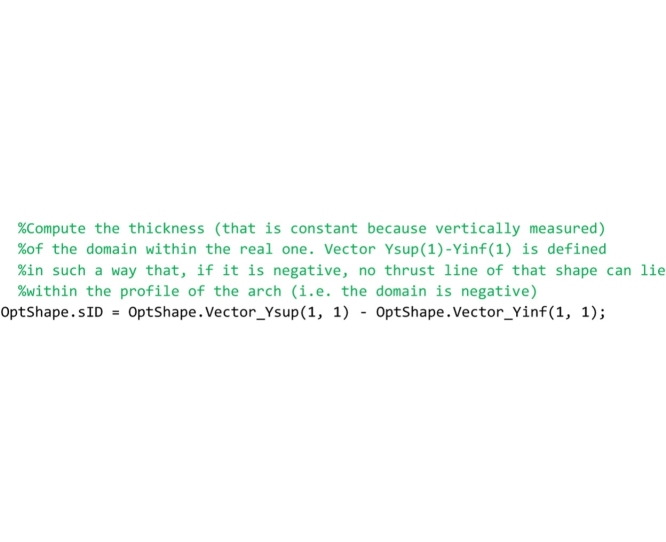

The last part of the code computes the safety factor. The minimum vertical thickness in correspondence to the action lines of the load vectors is computed and stored in the variable OptShape.RealArchMinimumThickness. Then, the full range factor of safety is computed and stored in the OptShape.Geom_Safety_Factor variable. The performance factor, that is the reciprocal of the full range factor, is not computed in this routine but it is calculated directly in the main routine and is showed in both the graphical output results and the legend.

**Figure:**
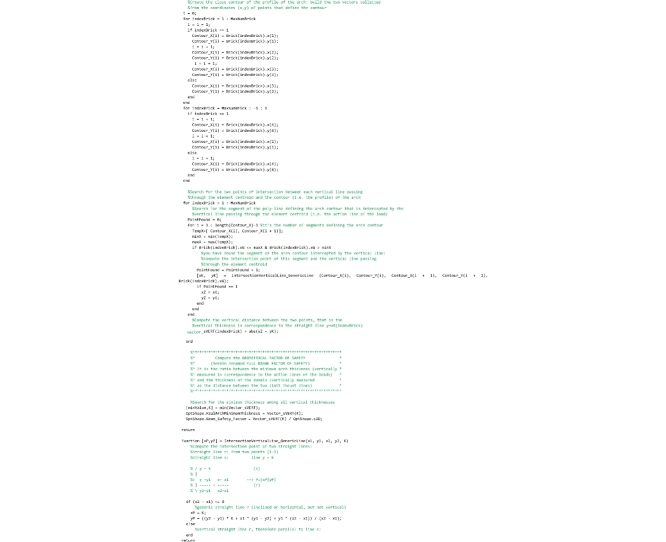

## The “GeomFactor” routine

This routine, that must be saved to the ‘GeomFactor.m’ file, computes the safety factor according to the original Heymanian theory based on the research of the arch of minimal thickness within the profile of the actual arch capable of supporting the same system of forces.

As for the “FullRangeFactor” routine above, after the global variables used herein are declared, the coordinates of the points of pressure in the right and left interfaces are computed.

**Figure:**
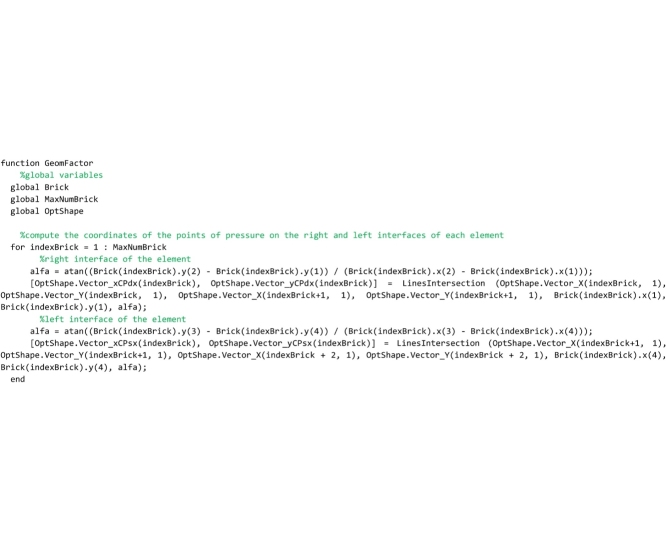

In the following lines, the thickness of the arch of minimal thickness within the real one is searched. To perform this search, two vectors are defined for each joint (i.e. for both the left and right interface of each element): vector oriented from the centroid of the joint to the point of pressure (referred to as Vector_dSUP in the code) and vector oriented from the point of pressure to the extrados (referred to as Vector_sSUP in the code) in the case in which the line of thrust is above the geometrical axis; vector oriented from the centroid of the joint to the point of pressure (referred to as Vector_dINF in the code) and vector oriented from the point of pressure to the intrados (referred to as Vector_sINF in the code) in the case in which the line of thrust is under the geometrical axis. The lines that follow perform this computation.

**Figure:**
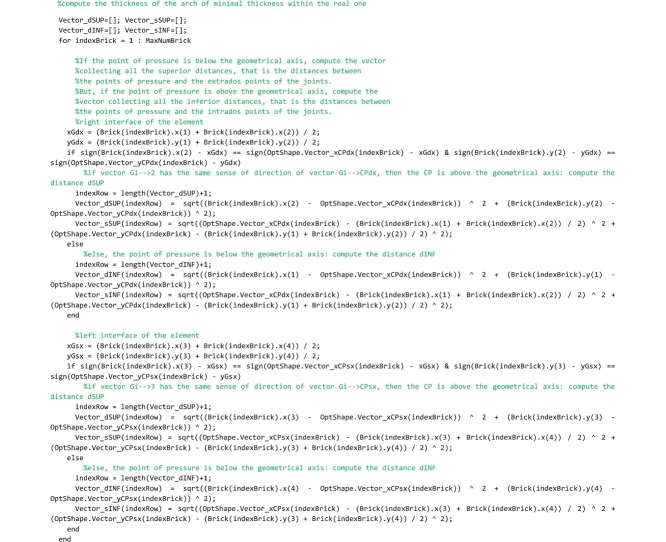

The lines that follow are to compute the longest superior thickness vector (maximum value of Vector_sSUP stored in the variable sSUPmax) and the longest inferior thickness vector (minimum value of Vector_sINF stored in the variable sINFmax) and they are assigned to the variables OptShape.sSUP and OptShape.sINF respectively. They are also used to compute the thickness of the ideal arch (OptShape.sID).

**Figure:**
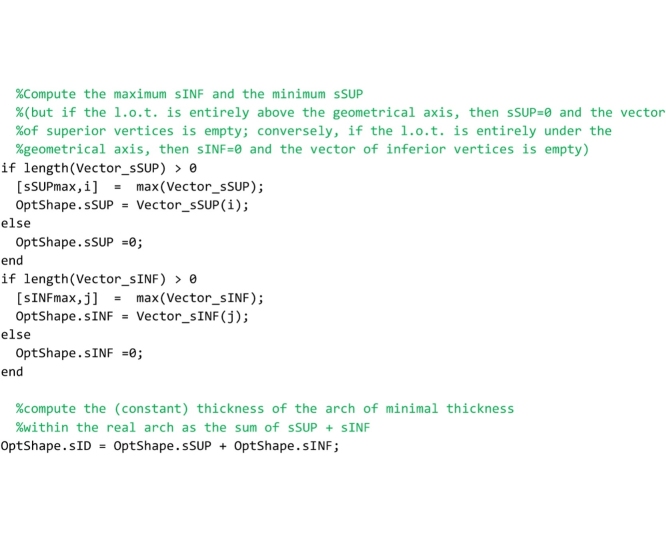

The last lines of the code are devoted to the computation of the geometrical factor of safety, obtained by the ratio between the minimum thickness of all joints and the thickness of the arch of minimal thickness within the actual one.

**Figure:**
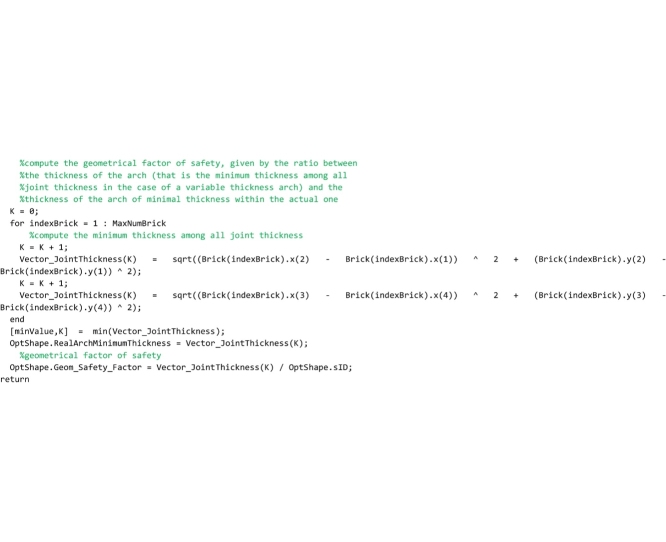

## The “LinesIntersection” and “OrientedLineAngle” functions

These two service functions complete the Matlab code and must be saved to the ‘LinesIntersection.m’ and ‘OrientedLineAngle.m’ files respectively. The “LinesIntersection” function is called both by the “FullRangeFactor” and “GeomFactor” routines to compute the coordinates of the points of pressure in the left and right interface of each joint. The coordinates of two points (x1,y1;x2,y2) belonging to the first straight line and a point (xO,yO) plus the inclination angle (alfa) of the second straight line are passed to this function. The function returns the coordinates of the intersection point ([xP,yP] in the code).

**Figure:**
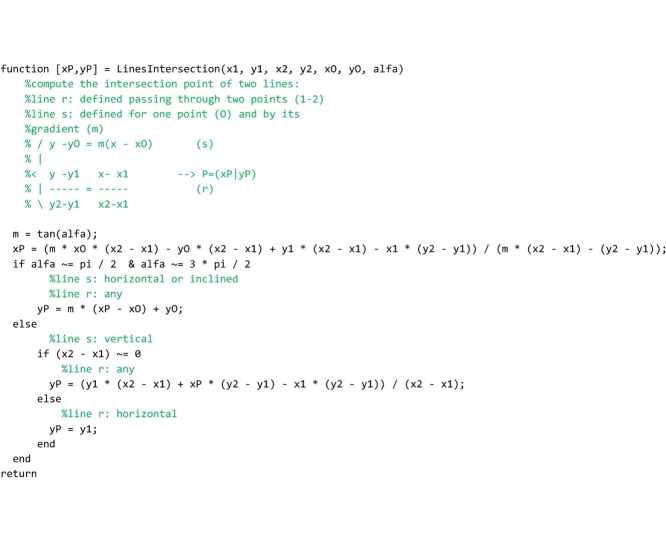

The “OrientedLineAngle” function is used by the main section of the program to plot the Heymanian arch of minimal thickness. The horizontal and vertical components of the direction vector of a straight line (DeltaX, DeltaY) are passed to the function that returns the inclination angle of that line in the interval [0-2Π].

**Figure:**