# Supplementary material for: The Matlab code of the method based on the Full Range Factor for assessing the safety of masonry arches
Source: MethodsX. 2019 Jun 4;6:1521–42. doi: 10.1016/j.mex.2019.05.033 (PMC6660453; doi:10.1016/j.mex.2019.05.033)
Supplement: Supplementary file 2 [file mmc2.docx]

**Matlab Code**

**FRS_Method.m**

%***********************************************

%* FRS Method *

%* ---------- *

%* Release: 1.0.0 – 2018-2019 *

%* Developed: Matlab v.6.1.0.450 Release 12.1 *

%* Authors: Stefano Galassi & Giacomo Tempesta *

%***********************************************

%Clear all variables from memory

clear all

%************************************

%Global variables

global ResearchLoadFACTOR

global Brick

Brick = struct('x',0,'y',0,'xG',0,'yG',0,'Weight',0,'Fy',0)

global VectorF

global MaxNumBrick

global OptShape

OptShape=struct('Type_Analysis',2,'LoadFactor',1,'Vector_Y',0,'Vector_X',0,'Vector_xCPdx',0,'Vector_yCPdx',0,'Vector_xCPsx',0,'Vector_yCPsx',0,'sSUP',0,'sINF',0,'sID',0,'RealArchMinimumThickness',0,'Geom_Safety_Factor',1,'Vector_Ysup',0,'Vector_Yinf',0,'ThrustH',0)

%************************************

%User Input Data

filename='RandomArch.txt' %name of the input file

Depth=100; %depth of the arch (cm)

UnitWeight=2000; %unit weight of the material (kgf/m^3)

MaxNumBrick=7; %number of bricks

OptShape.Type_Analysis=1; %1=FRS our modified method - 2=GFS by Heyman

ResearchLoadFACTOR=0; %0=NO - 1=YES

%************************************

fileID = fopen(filename,'r'); %load the geometry: name of the input file

%Lines beginning with % are not considered

[NumPunto,X,Y] = textread(filename,'%s%f%f','commentstyle','matlab');

fclose(fileID);

%Assign coordinates of element vertices to the variables

j=0;

for indexBrick = 1 : MaxNumBrick

for i = 1 : 4

j=j+1;

Brick(indexBrick).x(i)=X(j);

Brick(indexBrick).y(i)=Y(j);

Brick(indexBrick).Fy=0; %(kgf)

end

end

%Brick(3).Fy=-2000; %input an additional vertical force in correspondence to the third element (kgf)

%Compute the coordinates of element centroids

for indexBrick = 1 : MaxNumBrick

[Brick(indexBrick).xG,Brick(indexBrick).yG,Brick(indexBrick).Weight]=ComputeElementCentroid (Brick(indexBrick).x,Brick(indexBrick).y,Depth,UnitWeight);

end

%Compute the line of thrust closest to the geometrical axis and the domain of equilibrium (GFS o FRS)

RunAnalysis

%************

%* GRAPHICS *

%************

axis equal

grid on

xlabel('abscissae [cm]')

ylabel('ordinates [cm]')

%plot the elements of the arch

for indexBrick = 1 : MaxNumBrick

hold on

plot(Brick(indexBrick).x,Brick(indexBrick).y,'b') %border of elements (blue)

fill(Brick(indexBrick).x,Brick(indexBrick).y,'y') %fill of the elements (yellow)

hold on

scatter(Brick(indexBrick).xG,Brick(indexBrick).yG,2,'b') %element centroid (blue)

hold on

elementID = int2str(indexBrick);

text(Brick(indexBrick).xG,Brick(indexBrick).yG,elementID) %write the element number

end

%plot the line of thrust closest to the geometrical axis

hold on

ThrustLine=plot(OptShape.Vector_X,OptShape.Vector_Y,'r','linewidth',2); %red and thick thrust line

%plot the points of pressure in the right and left interfaces

hold on

scatter(OptShape.Vector_xCPdx, OptShape.Vector_yCPdx,3,'r') %points of pressure in the right interface (red)

hold on

scatter(OptShape.Vector_xCPsx, OptShape.Vector_yCPsx,3,'r') % points of pressure in the left interface (red)

%plot the safety domain (GFS or FRS)

if OptShape.Type_Analysis == 1 %"FullRangeFactor"

%Plot the two limit lines of thrust (tangent to the intrados and extrados)

%l.o.t. tangent to the intrados

Lbound=plot(OptShape.Vector_X,OptShape.Vector_Yinf,'b'); %upper bound (blue)

% l.o.t. tangent to the extrados

Ubound=plot(OptShape.Vector_X,OptShape.Vector_Ysup,'g'); %lower bound (green)

%Title and Legend

title('Full Range Factor of Safety')

legend([ThrustLine,Ubound,Lbound],'Thrust Line','Upper bound','Lower bound')

IdealArchThickness=strcat('Ideal Arch Thickness: ',num2str(OptShape.sID,'%.2f'),' cm');

text(min(xlim)+10,min(ylim)+130, IdealArchThickness)

FullRangeFactor=strcat('Full Range Factor of Safety (k): ',num2str(OptShape.Geom_Safety_Factor,'%.4f'));

text(min(xlim)+10,min(ylim)+100, FullRangeFactor)

PerformanceFactor=strcat('Performance Factor (1/k): ',num2str(1 / OptShape.Geom_Safety_Factor,'%.4f'));

text(min(xlim)+10,min(ylim)+70, PerformanceFactor)

if OptShape.sID < 0

text(min(xlim)+10,min(ylim)+40, 'No thrust line of this shape lies within the arch profile! Unsafe arch!')

end

else % 2 = "GeometrialFactorOfSafety"

%Plot the ideal arch within the real one, reducing the thickness of the arch.

%define the intrados and extrados curves of the arch

for i = 1 : MaxNumBrick

angleDX = OrientedLineAngle(Brick(i).x(2) - Brick(i).x(1), Brick(i).y(2) - Brick(i).y(1)); %right angle

angleSX = OrientedLineAngle(Brick(i).x(3) - Brick(i).x(4), Brick(i).y(3) - Brick(i).y(4)); %left angle

j=i+1;

UboundX(i) = (Brick(i).x(1) + Brick(i).x(2)) / 2 + OptShape.sSUP * cos(angleDX);

UboundX(j) = (Brick(i).x(3) + Brick(i).x(4)) / 2 + OptShape.sSUP * cos(angleSX);

UboundY(i) = (Brick(i).y(1) + Brick(i).y(2)) / 2 + OptShape.sSUP * sin(angleDX);

UboundY(j) = (Brick(i).y(3) + Brick(i).y(4)) / 2 + OptShape.sSUP * sin(angleSX);

LboundX(i) = (Brick(i).x(1) + Brick(i).x(2)) / 2 - OptShape.sINF * cos(angleDX);

LboundX(j) = (Brick(i).x(3) + Brick(i).x(4)) / 2 - OptShape.sINF * cos(angleSX);

LboundY(i) = (Brick(i).y(1) + Brick(i).y(2)) / 2 - OptShape.sINF * sin(angleDX);

LboundY(j) = (Brick(i).y(3) + Brick(i).y(4)) / 2 - OptShape.sINF * sin(angleSX);

end

Ubound=plot(UboundX,UboundY,'g'); %upper bound

Lbound=plot(LboundX,LboundY,'b'); %lower bound

%Title and Legend

title('Geometrical Factor of Safety')

legend([ThrustLine,Ubound,Lbound],'Thrust Line','Upper bound','Lower bound')

IdealArchThickness=strcat('Ideal Arch Thickness: ',num2str(OptShape.sID,'%.2f'),' cm');

text(min(xlim)+10,min(ylim)+130, IdealArchThickness)

GeometricalFactor=strcat('Geometrical Factor of Safety (k): ',num2str(OptShape.Geom_Safety_Factor,'%.4f'));

text(min(xlim)+10,min(ylim)+100, GeometricalFactor)

if OptShape.sID > OptShape.RealArchMinimumThickness

text(min(xlim)+10,min(ylim)+70, 'No thrust line of this shape lies within the arch profile! Unsafe arch!')

end

end

if ResearchLoadFACTOR == 1 %YES

Message=strcat('Collapse factor: ',num2str(OptShape.LoadFactor,'%.4f'));

text(min(xlim)+10,min(ylim)+10, Message)

end

**ComputeElementCentroid.m**

function [xG,yG,Weight] = ComputeElementCentroid(X,Y,Depth,UnitWeight)

%************

%* Area *

%************

Area = 0;

for i = 1 : 4

J = i + 1;

if i == 4

J = 1;

end

Area = Area + 0.5 * (Y(i) + Y(J)) * (X(J) - X(i));

end

%************************

%* First Moment of Area *

%* about the x axis *

%************************

Sx = 0;

for i = 1 : 4

J = i + 1;

if i == 4

J = 1;

end

Sx = Sx + (1 / 6) * (Y(i) ^ 2 + Y(i) * Y(J) + Y(J) ^ 2) * (X(J) - X(i));

end

%************************

%* First Moment of Area *

%* about the y axis *

%************************

Sy = 0;

for i = 1 : 4

J = i + 1;

if i == 4

J = 1;

end

Sy = Sy + (-1 / 6) * (X(i) ^ 2 + X(i) * X(J) + X(J) ^ 2) * (Y(J) - Y(i));

end

%*******************

%* Centroid G(x,y) *

%*******************

xG = Sy / Area;

yG = Sx / Area;

%*************************

%* Weight of the element *

%*************************

Weight = -Area * Depth * UnitWeight / 1000000;

return

**RunAnalysis.m**

function RunAnalysis

%global variables

global OptShape

global ResearchLoadFACTOR

TempLoadFACTOR = 1;

LambdaMin = 1; %value that verifies the arch (safe arch)

LambdaMax = 100000; %does not verify the arch (unsafe arch)

if OptShape.Type_Analysis == 1 %1 = 'FullRangeFactor' = our modified method (FRS)

while (LambdaMax - LambdaMin) >= 0.000000001 | OptShape.sID <= 0

if ResearchLoadFACTOR == 1 %YES

TempLoadFACTOR = (LambdaMin + LambdaMax) / 2;

end

%computes the line of thrust closet to the geometrical axis

BestThrustLine(TempLoadFACTOR)

%computes the Full Range Factor of Safety (our modified method - FRS)

FullRangeFactor

if TempLoadFACTOR == 1

break

end

if ResearchLoadFACTOR == 1 %YES

if OptShape.sID > 0 %safe arch

LambdaMin = TempLoadFACTOR;

else %unsafe arch

LambdaMax = TempLoadFACTOR;

end

end

end

else % 2 = 'GeometricalFactorOfSafety' = GFS method by Heyman

while (LambdaMax - LambdaMin) >= 0.000000001 | OptShape.sID >= OptShape.RealArchMinimumThickness

if ResearchLoadFACTOR == 1 %YES

TempLoadFACTOR = (LambdaMin + LambdaMax) / 2;

end

%computes the line of thrust closet to the geometrical axis

BestThrustLine(TempLoadFACTOR)

%computes the Geometrical Factor of Safety (GFS by Heyman)

GeomFactor

if TempLoadFACTOR == 1

break

end

if ResearchLoadFACTOR == 1 %YES

if OptShape.sID < OptShape.RealArchMinimumThickness %safe arch

LambdaMin = TempLoadFACTOR;

else %unsafe arch

LambdaMax = TempLoadFACTOR;

end

end

end

end

OptShape.LoadFactor = TempLoadFACTOR;

return

**BestThrustLine.m**

function BestThrustLine(TempLoadFACTOR)

global MaxNumBrick

global Brick

global VectorF

global OptShape

%compute the load vector {F}

ComputeLoadVectorF(TempLoadFACTOR)

%*********************************************************************************

% Search for the line of thrust closest to the geometrical axis of the arch,

% assuming the poly-line through the element centroids to be the axis of the arch

%*********************************************************************************

%Compute vector H collected from the horizontal distances hi between the action lines of the load vectors.

%Point A (h1) is the rightmost point of the arch and point B (h_last)the leftmost one.

TempAbutmentDX=[Brick(1).x(1), Brick(1).x(2)];

Vector_H(1) = max(TempAbutmentDX) - Brick(1).xG;

for i = 2 : MaxNumBrick

Vector_H(i) = Brick(i - 1).xG - Brick(i).xG;

end

TempAbutmentSX=[Brick(MaxNumBrick).x(3), Brick(MaxNumBrick).x(4)];

Vector_H(MaxNumBrick + 1) = Brick(MaxNumBrick).xG - min(TempAbutmentSX);

%build the general matrix [D], symmetric, with entries collected from vector {H}

for indexRow = 1 : MaxNumBrick

for indexColumn = indexRow : indexRow + 1

if indexRow == indexColumn

%entries of the main diagonal

Matrix_D(indexRow, indexColumn) = (Vector_H(indexRow) + Vector_H(indexRow + 1)) / (Vector_H(indexRow) * Vector_H(indexRow + 1));

else

if indexColumn > MaxNumBrick

break

end

%entries in the upper triangle

Matrix_D(indexRow, indexColumn) = -1 / Vector_H(indexColumn);

%entries in the lower triangle

Matrix_D(indexColumn, indexRow) = Matrix_D(indexRow, indexColumn);

end

end

end

%build the vertical load vector {T1}

%(it obtains the values from the load vector {F})

for indexRow = 1 : MaxNumBrick

Vector_T1(indexRow, 1) = VectorF(indexRow * 3 - 1);

end

%build vector {T2}, whose only nonzero entry is 1/h1 (i.e. the first entry)

Vector_T2 = zeros(MaxNumBrick,1);

Vector_T2(1, 1) = 1 / Vector_H(1);

%build vector {T3}, whose only nonzero entry is 1/h_last (i.e. the last entry)

Vector_T3 = zeros(MaxNumBrick,1);

Vector_T3(MaxNumBrick, 1) = 1 / Vector_H(length(Vector_H));

%compute the inverse of matrix [D]: [Dinv]

Matrix_Dinv = inv(Matrix_D);

% -1

%compute the product {R1} = [D] * {T1}

Vector_R1 = Matrix_Dinv * Vector_T1;

% -1

%compute the product {R2} = [D] * {T2}

Vector_R2 = Matrix_Dinv * Vector_T2;

% -1

%compute the product {R3} = [D] * {T3}

Vector_R3 = Matrix_Dinv * Vector_T3;

%build matrix [N], symmetric, with entries collected from vectors {R1},{R2},{R3}

Matrix_N = zeros(3,3);

SumR1 = 0; SumR2 = 0; SumR3 = 0;

SumR1R2 = 0; SumR1R3 = 0; SumR2R3 = 0;

for i = 1 : length(Vector_R1)

SumR1 = SumR1 + Vector_R1(i, 1) ^ 2;

SumR2 = SumR2 + Vector_R2(i, 1) ^ 2;

SumR3 = SumR3 + Vector_R3(i, 1) ^ 2;

SumR1R2 = SumR1R2 + Vector_R1(i, 1) * Vector_R2(i, 1);

SumR1R3 = SumR1R3 + Vector_R1(i, 1) * Vector_R3(i, 1);

SumR2R3 = SumR2R3 + Vector_R2(i, 1) * Vector_R3(i, 1);

end

Matrix_N(1, 1) = SumR1;

Matrix_N(2, 2) = SumR2;

Matrix_N(3, 3) = SumR3;

Matrix_N(1, 2) = SumR1R2;

Matrix_N(1, 3) = SumR1R3;

Matrix_N(2, 3) = SumR2R3;

Matrix_N(2, 1) = Matrix_N(1, 2);

Matrix_N(3, 1) = Matrix_N(1, 3);

Matrix_N(3, 2) = Matrix_N(2, 3);

%build vector {W} and collect it with the least squares entries

SumR1G = 0; SumR2G = 0; SumR3G = 0;

for i = 1 : MaxNumBrick

SumR1G = SumR1G + Vector_R1(i, 1) * Brick(i).yG;

SumR2G = SumR2G + Vector_R2(i, 1) * Brick(i).yG;

SumR3G = SumR3G + Vector_R3(i, 1) * Brick(i).yG;

end

Vector_W(1, 1) = SumR1G;

Vector_W(2, 1) = SumR2G;

Vector_W(3, 1) = SumR3G;

%compute the inverse of matrix [N]: [Ninv]

Matrix_Ninv = inv(Matrix_N);

% -1

%compute the product {P} = [N] * {W}

Vector_P = Matrix_Ninv * Vector_W;

%solve the matrix system {Y} = {R1}K + {R2}yA + {R3}yB

%and provide entries of vector {Y}, that is the heights (i.e. ordinates) of vertices of the line of thrust

OptShape. Vector_Y = zeros(length(Vector_R1),1);

for i = 1 : length(OptShape.Vector_Y)

OptShape.Vector_Y(i, 1) = Vector_R1(i, 1) * Vector_P(1, 1) + Vector_R2(i, 1) * Vector_P(2, 1) + Vector_R3(i, 1) * Vector_P(3, 1);

end

%input the first and last vertices of the line of thrust (points A and B) in vector {Y}

TempVector_Y = OptShape.Vector_Y;

OptShape.Vector_Y=[Vector_P(2, 1); TempVector_Y; Vector_P(3, 1)];

%store the value of the thrust H

OptShape.ThrustH = 1 / Vector_P(1, 1);

%compute vector {X}, that collects the abscissae of vertices of the line of thrust

OptShape.Vector_X=[max(TempAbutmentDX), Brick.xG, min(TempAbutmentSX)];

OptShape.Vector_X=OptShape.Vector_X';

return

**ComputeLoadVectorF.m**

function ComputeLoadVectorF(TempSafetyFACTOR)

%global variables

global Brick

global MaxNumBrick

global VectorF

for indexBrick = 1 : MaxNumBrick

%load vector {F}

%Horizontal forces Fx (Kgf): equal to zero

VectorF(indexBrick * 3 - 2) = 0;

%Vertical forces Fy (Kgf): weight of the element + additional vertical forces inputted by the user

VectorF(indexBrick * 3 - 1) = -(Brick(indexBrick).Weight - Brick(indexBrick).Fy * TempSafetyFACTOR) * sin(pi / 2 + pi);

%Moments (Kgf*cm): equal to zero

VectorF(indexBrick * 3) = 0;

end

return

**FullRangeFactor.m**

function FullRangeFactor

%global variables

global Brick

global MaxNumBrick

global OptShape

%compute the coordinates of the points of pressure in the right and left interfaces of each element

for indexBrick = 1 : MaxNumBrick

%right interface of the element

alfa = atan((Brick(indexBrick).y(2) - Brick(indexBrick).y(1)) / (Brick(indexBrick).x(2) - Brick(indexBrick).x(1)));

[OptShape.Vector_xCPdx(indexBrick), OptShape.Vector_yCPdx(indexBrick)] = LinesIntersection (OptShape.Vector_X(indexBrick, 1), OptShape.Vector_Y(indexBrick, 1), OptShape.Vector_X(indexBrick+1, 1), OptShape.Vector_Y(indexBrick+1, 1), Brick(indexBrick).x(1), Brick(indexBrick).y(1), alfa);

%left interface of the element

alfa = atan((Brick(indexBrick).y(3) - Brick(indexBrick).y(4)) / (Brick(indexBrick).x(3) - Brick(indexBrick).x(4)));

[OptShape.Vector_xCPsx(indexBrick), OptShape.Vector_yCPsx(indexBrick)] = LinesIntersection (OptShape.Vector_X(indexBrick+1, 1), OptShape.Vector_Y(indexBrick+1, 1), OptShape.Vector_X(indexBrick + 2, 1), OptShape.Vector_Y(indexBrick + 2, 1), Brick(indexBrick).x(4), Brick(indexBrick).y(4), alfa);

end

%*********************************************************************

%* Search for the LIMITING LOWER LINE OF THRUST: PF-a *

%* It is the line of thrust that is all above the intrados poly-line *

%*********************************************************************

for indexBrick = 1 : MaxNumBrick

%search for the segment of the thrust line that is intercepted by the vertical line

%passing through the intrados point of the right interface of the element

for i = 1 : length(OptShape.Vector_Y)-1 %number of segments of the thrust line

IntersecFound=0;

if Brick(indexBrick).x(1) <= OptShape.Vector_X(i, 1) & Brick(indexBrick).x(1) >= OptShape.Vector_X(i+1, 1)

%you have found the segment of the thrust line intercepted by the vertical line:

%compute the intersection point of this segment and the vertical line passing

%through the intrados vertex in the right interface of the element

[xK,yK] = IntersectionVerticalLine_GenericLine (OptShape.Vector_X(i, 1), OptShape.Vector_Y(i, 1), OptShape.Vector_X(i+1, 1), OptShape.Vector_Y(i+1, 1), Brick(indexBrick).x(1));

IntersecFound=1;

%now compute the vertical distance (may be positive or negative because it’s a vector)

%between the intersection point and the intrados point

Vector_dINF(indexBrick * 2 - 1) = Brick(indexBrick).y(1) - yK;

break

end

end

%if you have not found an intersection point, build an ideal point at infinity (negative)

if IntersecFound==0

Vector_dINF(indexBrick * 2 - 1) = 1E-24; %realmin

end

%search for the segment of the thrust line that is intercepted by the vertical line

%passing through the intrados point of the left interface of the element

for i = 1 : length(OptShape.Vector_Y)-1 % number of segments of the thrust line

IntersecFound=0;

if Brick(indexBrick).x(4) <= OptShape.Vector_X(i, 1) & Brick(indexBrick).x(4) >= OptShape.Vector_X(i+1, 1)

%you have found the segment of the thrust line intercepted by the vertical line:

%compute the intersection point of this segment and the vertical line passing

%through the intrados vertex in the left interface of the element

[xK,yK] = IntersectionVerticalLine_GenericLine (OptShape.Vector_X(i, 1), OptShape.Vector_Y(i, 1), OptShape.Vector_X(i+1, 1), OptShape.Vector_Y(i+1, 1), Brick(indexBrick).x(4));

IntersecFound=1;

%now compute the vertical distance (may be positive or negative because it’s a vector)

%between the intersection point and the intrados point

Vector_dINF(indexBrick * 2) = Brick(indexBrick).y(4) - yK;

break

end

end

%if you have not found an intersection point, build an ideal point at infinity (negative)

if IntersecFound==0

Vector_dINF(indexBrick * 2) = 1E-24; %realmin

end

end

%Compute the shortest distance vector, vertically arranged from

%point K of the thrust line to the intrados point of the arch

[dINFmax,j] = max(Vector_dINF); %it’s the minimum distance

OptShape.sINF = dINFmax;

%Compute the coordinates of the limit thrust line tangent to the intrados: PF-a

for i = 1 : length(OptShape.Vector_Y)

OptShape.Vector_Yinf(i, 1) = OptShape.Vector_Y(i, 1) + OptShape.sINF;

end

%*********************************************************************

%* Search for the LIMITING UPPER LINE OF THRUST: PF-b *

%* It is the line of thrust that is all under the extrados poly-line *

%*********************************************************************

for indexBrick = 1 : MaxNumBrick

%search for the segment of the thrust line that is intercepted by the vertical line

%passing through the extrados point of the right interface of the element

for i = 1 : length(OptShape.Vector_Y)-1 %number of segments of the thrust line

IntersecFound=0;

if Brick(indexBrick).x(2) <= OptShape.Vector_X(i, 1) & Brick(indexBrick).x(2) >= OptShape.Vector_X(i+1, 1)

%you have found the segment of the thrust line intercepted by the vertical line:

%compute the intersection point of this segment and the vertical line passing

%through the extrados vertex in the right interface of the element

[xK,yK] = IntersectionVerticalLine_GenericLine (OptShape.Vector_X(i, 1), OptShape.Vector_Y(i, 1), OptShape.Vector_X(i+1, 1), OptShape.Vector_Y(i+1, 1), Brick(indexBrick).x(2));

IntersecFound=1;

%now compute the vertical distance (may be positive or negative because it’s a vector)

%between the intersection point and the extrados point

Vector_dSUP(indexBrick * 2 - 1) = Brick(indexBrick).y(2) - yK;

break

end

end

%if you have not found an intersection point, build an ideal point at infinity (positive)

if IntersecFound==0

Vector_dSUP(indexBrick * 2 - 1) = 1E+24; %realmax

end

%search for the segment of the thrust line that is intercepted by the vertical line

%passing through the extrados point of the left interface of the element

for i = 1 : length(OptShape. Vector_Y)-1 %number of segments of the thrust line

IntersecFound=0;

if Brick(indexBrick).x(3) <= OptShape.Vector_X(i, 1) & Brick(indexBrick).x(3) >= OptShape.Vector_X(i+1, 1)

%you have found the segment of the thrust line intercepted by the vertical line:

%compute the intersection point of this segment and the vertical line passing

%through the extrados vertex in the left interface of the element

[xK, yK] = IntersectionVerticalLine_GenericLine (OptShape.Vector_X(i, 1), OptShape.Vector_Y(i, 1), OptShape.Vector_X(i+1, 1), OptShape.Vector_Y(i+1, 1), Brick(indexBrick).x(3));

IntersecFound=1;

%now compute the vertical distance (may be positive or negative because it’s a vector)

%between the intersection point and the extrados point

Vector_dSUP(indexBrick * 2) = Brick(indexBrick).y(3) - yK;

break

end

end

%if you have not found an intersection point, build an ideal point at infinity (positive)

if IntersecFound==0

Vector_dSUP(indexBrick * 2) = 1E+24; %realmax

end

end

%Compute the shortest distance vector, vertically arranged from

%point K of the thrust line to the extrados point of the arch

[dSUPmin,i] = min(Vector_dSUP);

OptShape.sSUP = dSUPmin;

%Compute the coordinates of the limit thrust line tangent to the extrados: PF-b

for i = 1 : length(OptShape.Vector_Y)

OptShape.Vector_Ysup(i, 1) = OptShape.Vector_Y(i, 1) + OptShape.sSUP;

end

%Compute the thickness (that is constant because vertically measured)

%of the domain within the real one. Vector Ysup(1)-Yinf(1) is defined

%in such a way that, if it is negative, no thrust line of that shape can lie

%within the profile of the arch (i.e. the domain is negative)

OptShape.sID = OptShape.Vector_Ysup(1, 1) - OptShape.Vector_Yinf(1, 1);

%Create the close contour of the profile of the arch: build the two vectors collected

%from the coordinates (x,y) of points that define the contour

i = 0;

for indexBrick = 1 : MaxNumBrick

i = i + 1;

if indexBrick == 1

Contour_X(i) = Brick(indexBrick).x(1);

Contour_Y(i) = Brick(indexBrick).y(1);

i = i + 1;

Contour_X(i) = Brick(indexBrick).x(2);

Contour_Y(i) = Brick(indexBrick).y(2);

i = i + 1;

Contour_X(i) = Brick(indexBrick).x(3);

Contour_Y(i) = Brick(indexBrick).y(3);

else

Contour_X(i) = Brick(indexBrick).x(3);

Contour_Y(i) = Brick(indexBrick).y(3);

end

end

for indexBrick = MaxNumBrick : -1 : 1

if indexBrick == 1

i = i + 1;

Contour_X(i) = Brick(indexBrick).x(4);

Contour_Y(i) = Brick(indexBrick).y(4);

i = i + 1;

Contour_X(i) = Brick(indexBrick).x(1);

Contour_Y(i) = Brick(indexBrick).y(1);

else

i = i + 1;

Contour_X(i) = Brick(indexBrick).x(4);

Contour_Y(i) = Brick(indexBrick).y(4);

end

end

%Search for the two points of intersection between each vertical line passing

%through the element centroid and the contour (i.e. the profile) of the arch

for indexBrick = 1 : MaxNumBrick

%Search for the segment of the poly-line defining the arch contour that is intercepted by the

%vertical line passing through the element centroid (i.e. the action line of the load)

PointFound = 0;

for i = 1 : length(Contour_X)-1 %it’s the number of segments defining the arch contour

TempX=[ Contour_X(i), Contour_X(i + 1)];

minX = min(TempX);

maxX = max(TempX);

if Brick(indexBrick).xG <= maxX & Brick(indexBrick).xG > minX

%you have found the segment of the arch contour intercepted by the vertical line:

%compute the intersection point of this segment and the vertical line passing

%through the element centroid

PointFound = PointFound + 1;

[xK, yK] = IntersectionVerticalLine_GenericLine (Contour_X(i), Contour_Y(i), Contour_X(i + 1), Contour_Y(i + 1), Brick(indexBrick).xG);

if PointFound == 1

xZ = xK;

yZ = yK;

end

end

end

%Compute the vertical distance between the two points, that is the

%vertical thickness in correspondence to the straight line y=xG(indexBrick)

Vector_sVERT(indexBrick) = abs(yZ - yK);

end

%******************************************************************

%* Compute the GEOMETRICAL FACTOR OF SAFETY *

%* (herein renamed FULL RANGE FACTOR OF SAFETY) *

%* It is the ratio between the minimum arch thickness (vertically *

%* measured in correspondence to the action lines of the loads) *

%* and the thickness of the domain (vertically measured *

%* as the distance between the two limit thrust lines) *

%******************************************************************

%Search for the minimum thickness among all vertical thicknesses

[minValue,K] = min(Vector_sVERT);

OptShape.RealArchMinimumThickness = Vector_sVERT(K);

OptShape.Geom_Safety_Factor = Vector_sVERT(K) / OptShape.sID;

return

function [xP,yP] = IntersectionVerticalLine_GenericLine(x1, y1, x2, y2, K)

%compute the intersection point of two straight lines:

%straight line r: from two points (1-2)

%straight line s: vertical line y = k

% / y = k (s)

% |

%< y -y1 x- x1 --> P=(xP|yP)

% | ----- = ----- (r)

% \ y2-y1 x2-x1

if (x2 - x1) ~= 0

%generic straight line r (inclined or horizontal, but not vertical)

xP = K;

yP = ((y2 - y1) * K + x1 * (y1 - y2) + y1 * (x2 - x1)) / (x2 - x1);

else

%vertical straight line r, therefore parallel to line s:

end

return

**GeomFactor.m**

function GeomFactor

%global variables

global Brick

global MaxNumBrick

global OptShape

%compute the coordinates of the points of pressure on the right and left interfaces of each element

for indexBrick = 1 : MaxNumBrick

%right interface of the element

alfa = atan((Brick(indexBrick).y(2) - Brick(indexBrick).y(1)) / (Brick(indexBrick).x(2) - Brick(indexBrick).x(1)));

[OptShape.Vector_xCPdx(indexBrick), OptShape.Vector_yCPdx(indexBrick)] = LinesIntersection (OptShape.Vector_X(indexBrick, 1), OptShape.Vector_Y(indexBrick, 1), OptShape.Vector_X(indexBrick+1, 1), OptShape.Vector_Y(indexBrick+1, 1), Brick(indexBrick).x(1), Brick(indexBrick).y(1), alfa);

%left interface of the element

alfa = atan((Brick(indexBrick).y(3) - Brick(indexBrick).y(4)) / (Brick(indexBrick).x(3) - Brick(indexBrick).x(4)));

[OptShape.Vector_xCPsx(indexBrick), OptShape.Vector_yCPsx(indexBrick)] = LinesIntersection (OptShape.Vector_X(indexBrick+1, 1), OptShape.Vector_Y(indexBrick+1, 1), OptShape.Vector_X(indexBrick + 2, 1), OptShape.Vector_Y(indexBrick + 2, 1), Brick(indexBrick).x(4), Brick(indexBrick).y(4), alfa);

end

%compute the thickness of the arch of minimal thickness within the real one

Vector_dSUP=[]; Vector_sSUP=[];

Vector_dINF=[]; Vector_sINF=[];

for indexBrick = 1 : MaxNumBrick

%If the point of pressure is below the geometrical axis, compute the vector

%collecting all the superior distances, that is the distances between

%the points of pressure and the extrados points of the joints.

%But, if the point of pressure is above the geometrical axis, compute the

%vector collecting all the inferior distances, that is the distances between

%the points of pressure and the intrados points of the joints.

%right interface of the element

xGdx = (Brick(indexBrick).x(1) + Brick(indexBrick).x(2)) / 2;

yGdx = (Brick(indexBrick).y(1) + Brick(indexBrick).y(2)) / 2;

if sign(Brick(indexBrick).x(2) - xGdx) == sign(OptShape.Vector_xCPdx(indexBrick) - xGdx) & sign(Brick(indexBrick).y(2) - yGdx) == sign(OptShape.Vector_yCPdx(indexBrick) - yGdx)

%if vector Gi-->2 has the same sense of direction of vector Gi-->CPdx, then the CP is above the geometrical axis: compute the distance dSUP

indexRow = length(Vector_dSUP)+1;

Vector_dSUP(indexRow) = sqrt((Brick(indexBrick).x(2) - OptShape.Vector_xCPdx(indexBrick)) ^ 2 + (Brick(indexBrick).y(2) - OptShape.Vector_yCPdx(indexBrick)) ^ 2);

Vector_sSUP(indexRow) = sqrt((OptShape.Vector_xCPdx(indexBrick) - (Brick(indexBrick).x(1) + Brick(indexBrick).x(2)) / 2) ^ 2 + (OptShape.Vector_yCPdx(indexBrick) - (Brick(indexBrick).y(1) + Brick(indexBrick).y(2)) / 2) ^ 2);

else

%else, the point of pressure is below the geometrical axis: compute the distance dINF

indexRow = length(Vector_dINF)+1;

Vector_dINF(indexRow) = sqrt((Brick(indexBrick).x(1) - OptShape.Vector_xCPdx(indexBrick)) ^ 2 + (Brick(indexBrick).y(1) - OptShape.Vector_yCPdx(indexBrick)) ^ 2);

Vector_sINF(indexRow) = sqrt((OptShape.Vector_xCPdx(indexBrick) - (Brick(indexBrick).x(1) + Brick(indexBrick).x(2)) / 2) ^ 2 + (OptShape.Vector_yCPdx(indexBrick) - (Brick(indexBrick).y(1) + Brick(indexBrick).y(2)) / 2) ^ 2);

end

%left interface of the element

xGsx = (Brick(indexBrick).x(3) + Brick(indexBrick).x(4)) / 2;

yGsx = (Brick(indexBrick).y(3) + Brick(indexBrick).y(4)) / 2;

if sign(Brick(indexBrick).x(3) - xGsx) == sign(OptShape.Vector_xCPsx(indexBrick) - xGsx) & sign(Brick(indexBrick).y(3) - yGsx) == sign(OptShape.Vector_yCPsx(indexBrick) - yGsx)

%if vector Gi-->3 has the same sense of direction of vector Gi-->CPsx, then the CP is above the geometrical axis: compute the distance dSUP

indexRow = length(Vector_dSUP)+1;

Vector_dSUP(indexRow) = sqrt((Brick(indexBrick).x(3) - OptShape.Vector_xCPsx(indexBrick)) ^ 2 + (Brick(indexBrick).y(3) - OptShape.Vector_yCPsx(indexBrick)) ^ 2);

Vector_sSUP(indexRow) = sqrt((OptShape.Vector_xCPsx(indexBrick) - (Brick(indexBrick).x(3) + Brick(indexBrick).x(4)) / 2) ^ 2 + (OptShape.Vector_yCPsx(indexBrick) - (Brick(indexBrick).y(3) + Brick(indexBrick).y(4)) / 2) ^ 2);

else

%else, the point of pressure is below the geometrical axis: compute the distance dINF

indexRow = length(Vector_dINF)+1;

Vector_dINF(indexRow) = sqrt((Brick(indexBrick).x(4) - OptShape.Vector_xCPsx(indexBrick)) ^ 2 + (Brick(indexBrick).y(4) - OptShape.Vector_yCPsx(indexBrick)) ^ 2);

Vector_sINF(indexRow) = sqrt((OptShape.Vector_xCPsx(indexBrick) - (Brick(indexBrick).x(3) + Brick(indexBrick).x(4)) / 2) ^ 2 + (OptShape.Vector_yCPsx(indexBrick) - (Brick(indexBrick).y(3) + Brick(indexBrick).y(4)) / 2) ^ 2);

end

end

%Compute the maximum sINF and the minimum sSUP

%(but if the l.o.t. is entirely above the geometrical axis, then sSUP=0 and the vector

%of superior vertices is empty; conversely, if the l.o.t. is entirely under the

%geometrical axis, then sINF=0 and the vector of inferior vertices is empty)

if length(Vector_sSUP) > 0

[sSUPmax,i] = max(Vector_sSUP);

OptShape.sSUP = Vector_sSUP(i);

else

OptShape.sSUP =0;

end

if length(Vector_sINF) > 0

[sINFmax,j] = max(Vector_sINF);

OptShape.sINF = Vector_sINF(j);

else

OptShape.sINF =0;

end

%compute the (constant) thickness of the arch of minimal thickness

%within the real arch as the sum of sSUP + sINF

OptShape.sID = OptShape.sSUP + OptShape.sINF;

%compute the geometrical factor of safety, given by the ratio between

%the thickness of the arch (that is the minimum thickness among all

%joint thickness in the case of a variable thickness arch) and the

%thickness of the arch of minimal thickness within the actual one

K = 0;

for indexBrick = 1 : MaxNumBrick

%compute the minimum thickness among all joint thickness

K = K + 1;

Vector_JointThickness(K) = sqrt((Brick(indexBrick).x(2) - Brick(indexBrick).x(1)) ^ 2 + (Brick(indexBrick).y(2) - Brick(indexBrick).y(1)) ^ 2);

K = K + 1;

Vector_JointThickness(K) = sqrt((Brick(indexBrick).x(3) - Brick(indexBrick).x(4)) ^ 2 + (Brick(indexBrick).y(3) - Brick(indexBrick).y(4)) ^ 2);

end

[minValue,K] = min(Vector_JointThickness);

OptShape.RealArchMinimumThickness = Vector_JointThickness(K);

%geometrical factor of safety

OptShape.Geom_Safety_Factor = Vector_JointThickness(K) / OptShape.sID;

return

**LinesIntersection.m**

function [xP,yP] = LinesIntersection(x1, y1, x2, y2, xO, yO, alfa)

%compute the intersection point of two lines:

%line r: defined passing through two points (1-2)

%line s: defined for one point (O) and by its

%gradient (m)

% / y -yO = m(x - xO) (s)

% |

%< y -y1 x- x1 --> P=(xP|yP)

% | ----- = ----- (r)

% \ y2-y1 x2-x1

m = tan(alfa);

xP = (m * xO * (x2 - x1) - yO * (x2 - x1) + y1 * (x2 - x1) - x1 * (y2 - y1)) / (m * (x2 - x1) - (y2 - y1));

if alfa ~= pi / 2 & alfa ~= 3 * pi / 2

%line s: horizontal or inclined

%line r: any

yP = m * (xP - xO) + yO;

else

%line s: vertical

if (x2 - x1) ~= 0

%line r: any

yP = (y1 * (x2 - x1) + xP * (y2 - y1) - x1 * (y2 - y1)) / (x2 - x1);

else

%line r: horizontal

yP = y1;

end

end

return

**OrientedLineAngle.m**

function [LineAngle] = OrientedLineAngle(DeltaX, DeltaY)

%**********************************************

%* returns the positive angle of an oriented *

%* straight line in the interval 0 --- 2PI. *

%**********************************************

%DeltaX and DeltaY are: the difference between the coordinates

%of the end point and the start point of the direction vector

%of a straight line.

if DeltaY > 0

if DeltaX > 0

LineAngle = atan(DeltaY / DeltaX);

elseif DeltaX < 0

LineAngle = atan(DeltaY / DeltaX);

LineAngle = pi - (-LineAngle);

elseif DeltaX == 0

LineAngle = pi / 2;

end

elseif DeltaY < 0

if DeltaX > 0

LineAngle = atan(DeltaY / DeltaX);

LineAngle = 2 * pi - (-LineAngle);

elseif DeltaX < 0

LineAngle = atan(DeltaY / DeltaX);

LineAngle = pi + LineAngle;

elseif DeltaX == 0

LineAngle = 3 * pi / 2;

end

elseif DeltaY == 0

if DeltaX > 0

LineAngle = 0;

elseif DeltaX < 0

LineAngle = pi;

end

end

return
